# Cutting soft materials: how material differences shape the response

**DOI:** 10.1038/s41524-025-01869-y

**Published:** 2026-01-06

**Authors:** Miguel Angel Moreno-Mateos, Paul Steinmann

**Affiliations:** 1 Institute of Applied Mechanics, Friedrich-Alexander-Universität Erlangen-Nürnberg, Egerlandstr. 5, 91058 Erlangen, Germany; 2 Glasgow Computational Engineering Centre, School of Engineering, University of Glasgow, G12 8QQ, Glasgow, United Kingdom

**Keywords:** Engineering, Materials science

## Abstract

Cutting soft materials is a complex process governed by the interplay of bulk large deformation, interfacial soft fracture, and contact forces with the cutting tool. Existing experimental characterizations and numerical models often fail to capture the variety of observed cutting behaviors, especially the transition from indentation to cutting and the roles of dissipative mechanisms. Here, we combine novel experimental cutting tests on three representative materials—a soft hydrogel, an elastomer, and food materials—with a coupled computational model that integrates soft fracture, adhesion, and frictional interactions. Our experiments reveal material-dependent cutting behaviors, with abrupt or smooth transitions from indentation to crack initiation, followed by distinct steady cutting regimes. The computational model captures these behaviors and shows that adhesion and damping contributions in the cohesive forces dominate tangential stresses, while Coulomb friction plays a negligible role due to low contact pressures. Together, these results provide new mechanistic insights into the physics of soft cutting and offer a unified framework for soft cutting mechanics to guide the design of soft materials, cutting tools, and cutting protocols, with direct relevance to surgical dissection and the engineering of food textures optimized for mastication.

## Introduction

Cutting is a fundamental process in both nature and technology, from cutting food in carnivores and herbivores^1,2^, insects slicing through plants^3,4^, package opening^5^, metal, rock, and wood cutting^6–8^, to robotic tools dissecting soft tissues^9^, insertion of needles^10–12^, or food products^13,14^. Yet, when it comes to soft solids, the mechanics of cutting remain poorly understood. Unlike brittle or hard materials, soft solids exhibit large deformations, rate-dependent dissipation, frictional sliding, adhesion, and even wear at the tool interface, all of which shape the transition from indentation to fracture^12,15–17^. Despite the prevalence of soft cutting in areas such as biomedical engineering^18–21^, food processing^22–24^, and soft robotics, a general framework capturing the physics of soft cutting is still missing.

Understanding this process raises several open questions: How does the initial indentation relate to the onset of cutting? What is the role of the material’s constitutive behavior in determining the cutting force? How do adhesion, wear, and Coulomb friction compete in controlling the cutting resistance? These questions challenge traditional models of fracture and friction^25^. Classical fracture mechanics^26^ and cohesive zone models typically assume sharp crack tips and separation governed by stress intensity or traction-separation laws. Yet, in cutting soft solids, experiments show that failure may initiate under a blunted blade through a progressive, geometry- and material-dependent process^27–31^. Together with numerical simulations, recent studies demonstrated that cutting produces a stronger strain localization than fracture^32,33^. This process has also been connected to the microstructure in elastomer and rubber materials^34^. Recent studies describe the fracture mechanics of polymer and composite structures across scales and related design pathways to enhance their failure response^35–40^. Analytic results connect stress intensity factors with tip sharpness and predict whether a crack propagates autonomously or remains in contact with the cutting tool. Computational and theoretical studies have begun to explore cohesive fracture and crack evolution in soft solids under localized and multiaxial loading^41–44^, laying important groundwork for understanding cutting as a soft fracture process.

The transition from stable indentation to unstable soft fracture is not purely governed by bulk toughness but modulated by viscous dissipation, adhesive interactions at the tool-material interface, and material compressibility. Although experimental setups have been proposed to investigate cutting in the absence of friction^45,46^, this is not representative of most cutting processes, where a cutting tool remains in contact with the material as it penetrates it^47,48^. In this context, surface microtexturing of cutting tools has been demonstrated to modulate friction in surgical cutting, improving efficiency and performance^49^. Moreover, materials of similar bulk stiffness can behave very differently under cutting. Moist food materials, for instance, exhibit cutting forces inconsistent with purely elastic or brittle theories; instead, rate-dependent and viscous failure modes are more accurate descriptors^50–52^. Double-network hydrogels exhibit extreme cutting toughness^53^. Cryogenic cutting of elastomers alters their constitutive behavior to enhance cuttability, effectively shifting the material response toward conditions optimal for cutting^54^. This raises fundamental questions about how energy is partitioned between elastic storage, fracture creation, and dissipative pathways–questions that traditional theories cannot fully answer.

In this work, we combine cutting experiments and a numerical model based on a cohesive zone with a contact approach to investigate how soft materials fail under a moving blade. Our model mimics three-dimensional cutting experiments explicitly accounting for adhesion, friction, and pseudo-viscous dissipation. This provides a mechanistic framework that goes beyond classical approaches. In particular, our unified framework substantiates specific hypotheses that lead to four central findings: the material and dissipative parameters directly modulate unstable/stable cutting transition; cutting initiates in the center beneath the blade; adhesion, rather than Coulomb friction, governs tangential forces during cutting; and cutting of processed food materials can be attributed to the damping effects arising from their complex internal structures.

Together, these findings offer a new understanding of soft cutting as a coupled problem of material response, interface mechanics, and energy dissipation. Our results clarify why nominally similar materials respond differently to the same blade and provide general principles applicable to biological, synthetic, and food-based soft solids. By bridging experiment and modeling, we propose a unified approach to a classically fragmented problem–shedding light on one of the most common yet least understood physical processes in soft matter.

## Results

### Material behavior, fracture, and friction govern cutting in soft materials

**Fig. 1:**
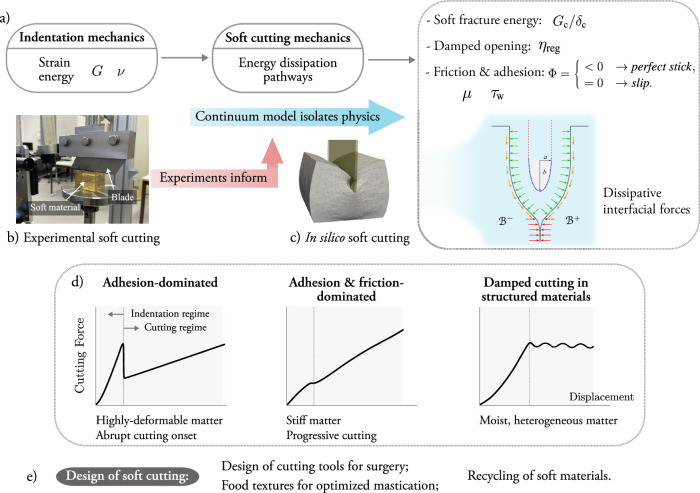
Overview of cutting mechanics in soft materials. **a** Schematic illustration of the energy pathways in soft material cutting, including initial indentation, fracture propagation, and dissipation mechanisms. Strain energy stored during indentation is dissipated through the creation of new surfaces (fracture energy), damping cohesive forces, and interfacial mechanisms such as friction and adhesion at the tool-material interface. The parameters of the unified model for soft cutting are direct descriptors of the cutting mechanisms, as described in Methods, Table 1. **b** Cutting experiments on soft materials reveal physical responses that inform **c** a coupled continuum model based on cohesive zone and contact mechanics, allowing the isolation of dominant mechanisms. The schematic highlights the balance of normal and tangential interfacial forces at the blade-material interface. **d** Representative force-displacement responses illustrate three major mechanisms in soft cutting mechanics: adhesion-dominated cutting, adhesion-friction coupled cutting, and damped cutting in materials with heterogeneous internal structures. **e** Application to design of soft cutting for surgical tools and food texture engineering design for optimized mastication.

Soft materials exhibit complex mechanical responses when cut, involving a combination of bulk material deformation, fracture decohesion at the cutting interface, and frictional interactions with the cutting tool. As illustrated in Fig. 1a, the strain energy accumulated in the bulk material during the initial phase of deformation (indentation) is released and dissipated to drive the formation of new cutting surfaces and to overcome frictional resistance arising from contact with the cutting tool. Understanding how these mechanisms interplay is essential to predict and control cutting behavior, yet it remains challenging due to their coupled, nonlinear nature.

**Fig. 2:**
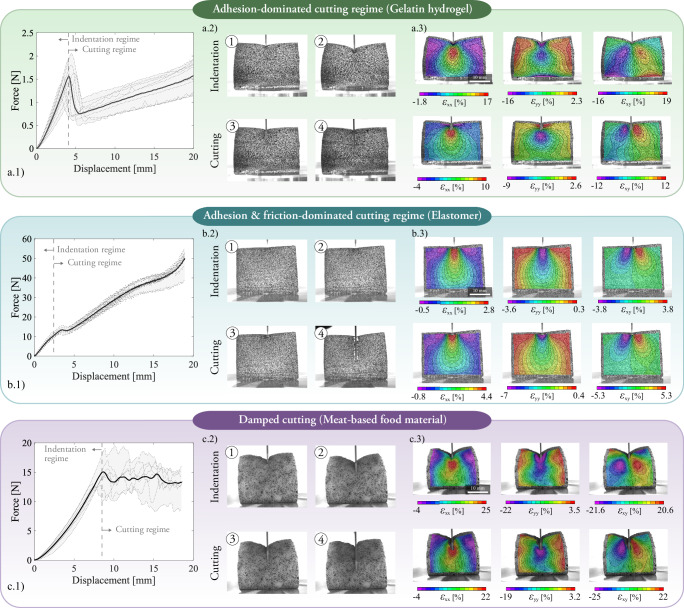
Experimental results for cutting tests. Three cutting mechanisms---adhesion-dominated, adhesion- & friction-dominated, and damped—are exemplified with gelatin hydrogel, elastomer, and meat-based samples. **a**–**c.1** Cutting force versus indentation curves. Ten repetitions are performed under the same test conditions. **a**–**c.2** Images of the surface of the samples during the indentation and the cutting regimes. The images correspond to the following values of displacement: for the hydrogel, 2.70 mm, 5.24 mm, 6.18 mm, and 14.14 mm; for the elastomer, 0.62 mm, 1.56 mm, 2.29 mm, and 14.14 mm; and for the meat-based food material, 6.24 mm, 9.76 mm, 10.40 mm, and 14.14 mm. **a**–**c.3** Surface engineering strain fields—defined according to Method 4.3—for one of the experimental repetitions (one with a force—displacement curve close to the mean one is selected). Three stages are analyzed, i.e., two before cutting onset—the second one at the end of the initial indentation—and one after the onset of cutting. The three stages correspond to the following values of displacement: for the hydrogel, 2.7 mm, 4.19 mm, and 6.17 mm; for the elastomer, 0.62 mm, 1.56 mm, and 2.29 mm; and for the meat-based sample, 4.80 mm, 9.76 mm, and 10.71 mm.

We identify and describe three distinct cutting behaviors across the tested materials—gelatin hydrogel, elastomer, and meat-based food material—that arise from the coupled effects of the material response at finite strains, the decohesion process along the cutting surface, and the frictional interaction between the material and the cutting tool. To that end, we leverage a custom-made experimental setup for the soft cutting experiment (see Figs. 1b and S1a). Additionally, we characterize the behavior of the materials under tensile deformation in Fig. S5. The cutting force versus displacement curves in Fig. 2 display different responses at the initial indentation regime ante cutting onset and at the posterior cutting regime.

The gelatin hydrogel exhibits an abrupt indentation-to-cutting transition characterized by a significant release of bulk strain energy, as illustrated in Fig. 2a. As a physically crosslinked polymer network, gelatin resists deformation through reversible physical bonds that stretch and accumulate energy as the blade compresses the material. The cutting force peaks early, ~4 mm of displacement, then drops sharply, and subsequently increases linearly. At a critical stress level, the crosslinked network fails abruptly -particularly near the blade tip-leading to what we denote as a cohesive collapse. This sharp drop in force reflects the sudden rupture of the material’s internal resistance. Interestingly, this behavior resembles that of materials with a hard outer shell or skin, where initial penetration is resisted until the blade breaks through, exposing a softer inner core. Following this rupture, the cutting force increases again, driven by frictional resistance as the blade interacts with the inner bulk. This friction is primarily adhesive in nature rather than due to Coulomb sliding, as it will be demonstrated by the computational model in the subsequent sections.

The elastomer displays a smooth, nearly linear indentation-to-cutting transition, characterized by a gradual increase in force with displacement and no distinct fracture event, as shown in Fig. 2b. Unlike the gelatin hydrogel, where cutting is accompanied by a sudden release of stored energy, the elastomer resists cutting progressively. This behavior reflects its high toughness relative to the other materials: the blade advances through steady energy input, without abrupt failure or cohesive collapse. The absence of a sharp transition suggests that the material undergoes distributed deformation and energy dissipation, consistent with high tangential friction forces.

Lastly, the meat-based food material, composed of finely ground meat and fat emulsified with binders such as starch, carrageenan, or soy protein, exhibits a smooth initial rise in cutting force, peaking ~8 mm–10 mm of blade displacement, followed by a fluctuating plateau at ~12 N to 16 N (see Fig. 2c). The non-increasing cutting force in the cutting regime indicates that tangential stresses are negligible; otherwise, they would increase with the enlarging interface area. This is consistent with the presence of grease in the processed food, which acts as a lubricant. Unlike brittle or highly crosslinked materials, there is no substantial force drop following the peak. Instead, the cutting force stabilizes, indicating a steady-state cutting regime. Internal heterogeneous structures in the material imply that cutting initiation is not simultaneous along the entire front; instead, the heterogeneous structure dampens and delays the local onset of fracture. This behavior is likely characteristic of processed sausage products, which are typically wet and cohesive. The absence of a sharp drop in force at the onset of cutting reflects the absence of a brittle fracture mechanism. Cutting initiates through ductile tearing and material flow, with no distinct transition between indentation and cutting, resulting in a gradual and continuous force response. The smooth transition, lacking a clear peak-drop-plateau sequence as observed in the hydrogel, is likely due to complex internal structures that damp the opening of the cutting surface (e.g., agglomerations of grease and meat in the processed material). This interpretation is supported by the serration patterns observed experimentally (cf. Fig. 2c.1) along the plateau phase. Internal heterogeneous structures intermittently affect resistance as the blade progresses. A final relevant observation is that once cutting is complete, the two halves of the specimen return to their reference configuration (cf. Movie S3), indicating that no major irreversible damage occurs.

The reader may note that bulk viscoelasticity is unlikely given the sufficiently low blade velocity that minimizes inelastic deformation of the bulk. A distinction between fracture energy and viscous dissipation is meaningful if the latter arises from relaxation processes well away from the crack tip. In cutting, however, where the blade and crack tip are in close proximity, it seems unlikely that major deformations occur far from the tip. While our study focuses on low blade velocities, we recognize that a detailed investigation of rate-dependent cutting in viscoelastic materials remains an important direction for future work.

We illustrate the surface strain fields during cutting in Fig. 2a–c.3 and with supporting videos in Movies S1, S2, and S3. Furthermore, we illustrate the three cutting mechanisms with cutting characterizations of additional food materials, presented in Section S3 and Fig. S4. The cutting responses of cheese, tofu, and marshmallow are discussed, highlighting the potential for engineering food textures optimized for mastication.

### A coupled computational model disentangles physical contributions in soft cutting

The analysis of the experimental results presented in the previous section faces limitations when testing specific hypotheses about the cutting mechanism as outlined in Fig. 1d. In particular, experimental techniques struggle to disentangle the contributions of tangential frictional stress—arising from contact pressure—from those of shear stress associated with wear. More broadly, the cohesive and frictional forces involved in cutting are intricately coupled and governed by the unknown constitutive behavior of the soft material. To address these challenges, we introduce a continuum-based computational model for the cutting of soft matter that replicates the experiments described in the previous sections (see the model in Methods).

Serving as a virtual testbed, our continuum model enables the systematic isolation of individual physical contributions: the material’s constitutive response (strain energy), the normal separation and the subsequent fracture-related energy dissipation at the cut surface due to contact with the cutting tool (see illustration in Fig. 1c and in Fig. S1c for more detail), and the tangential friction dissipation resulting from relative motion, which combines effects from contact pressure, and pressure-independent adhesive debonding and wear, as outlined in Fig. 1a. The model enables direct simulation of indentation, fracture onset, and progressive cutting under realistic conditions. Unlike frameworks that rely solely on stress thresholds to predict where cutting should initiate, we believe that our model directly simulates the physical process of soft fracture (surface separation) through cohesive zone modeling. The model is modular in structure: it integrates cohesive debonding along the cutting surface^55,56^, a rate-dependent regularization of surface opening, and shear stresses arising from both friction and adhesion^57^. These mechanisms are coupled within a unified framework, enabling systematic parametric studies in which specific contributions can be selectively activated or deactivated. This modularity is essential for understanding the complex physical interactions that govern the cutting process in soft materials.

**Fig. 3:**
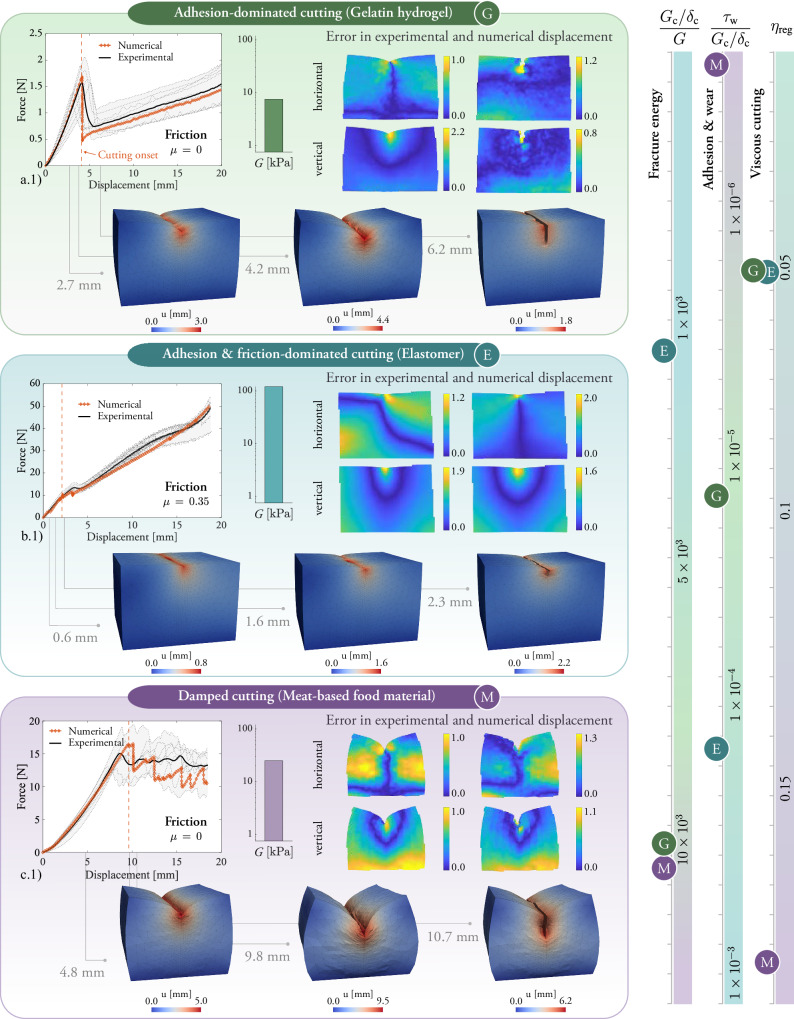
Virtual experiments were conducted with the computational framework for cutting soft materials. **a**–**c.1** Numerical and experimental cutting force versus displacement curves for the gelatin hydrogel, elastomer, and meat-based food material. **a**–**c.2** Stiffness of the sample as the parameter of the neo-Hookean model. **a**–**c.3** Relative error fields of the virtual experiments. The relative error field normalized by the average numerical displacement is computed as $| {u}_{x,\exp }-{u}_{x\rm{,num}}| /[{N}^{-1}{\sum }_{1}^{N}| {u}_{x\rm{,num}}| ]$ [−] and $| {u}_{y,\exp }-{u}_{y\rm{,num}}| /[{N}^{-1}{\sum }_{1}^{N}| {u}_{y\rm{,num}}| ]$ [–], and computed for three steps along the cutting process: two during indentation---before the onset of cutting—and one in the cutting regime. **a**–**c.4** Frames of the deformed samples along indentation and cutting in the virtual experiments for the three materials. The cutting model is calibrated with the material parameters, fracture, friction, and viscous parameters in Table 2. All samples have the same cutting length (in the direction of the cutting tool) of 30 mm and height of 21 mm. The gelatin hydrogel and elastomer samples have a width of 30 mm, while the meat-based samples have a width of 25 mm. The error fields are shown for one representative of all experimental repetitions.

As shown in Fig. 3, the force-displacement curves from the virtual experiments accurately capture the distinct cutting responses observed in the physical tests, remaining within the bounds of experimental variability. For the gelatin hydrogel, the sharp drop in cutting force ~4 mm of displacement reflects the sudden energy release associated with the indentation-to-cutting transition. In contrast, the elastomer exhibits a smooth transition with little to no reduction in cutting force, while the meat-based material shows a pronounced force plateau, consistent with experimental observations, following the onset of cutting. Moreover, the relative error between the experimentally measured and numerically simulated surface displacement fields confirms the overall strong agreement in deformation patterns between real and virtual experiments. The serrated shape of the force-displacement curve in the cutting regime is potentially related to structural inhomogeneities in the material.

To interpret the experimental observations across the three distinct cutting behaviors, we rely on a reduced set of model parameters, each directly associated with a specific physical mechanism, as summarized in Tables 1 and 2. After calibrating the model to reproduce the cutting response, we define three key dimensionless parameters, together with a viscosity parameter, that capture the underlying mechanics: $\frac{{G}_{\rm{c}}/{\delta }_{\rm{c}}}{G}$, the ratio of cohesive debonding energy to the material’s strain energy, governs the indentation-to-cutting transition and the steady-state cutting force; $\frac{{\tau }_{\rm{w}}}{{G}_{\rm{c}}/{\delta }_{\rm{c}}}$ characterizes the contact shear stress from combined adhesion and wear relative to the cutting force; *μ* represents the coefficient of Coulomb friction; and *η*_reg_ (in seconds) describes the rate-dependent, damped debonding of the cutting surface. The gelatin hydrogel is modeled with intermediate values $\frac{{G}_{\rm{c}}/{\delta }_{\rm{c}}}{G}=1.01\times 1{0}^{4}$ and $\frac{{\tau }_{\rm{w}}}{{G}_{\rm{c}}/{\delta }_{\rm{c}}}=1.06\times 1{0}^{-5}$, no Coulomb friction (*μ* = 0), and low viscosity *η*_reg_ = 0.05 s. The elastomer is described by a lower energy ratio $\frac{{G}_{\rm{c}}/{\delta }_{\rm{c}}}{G}=1.79\times 1{0}^{3}$, a higher adhesion ratio $\frac{{\tau }_{\rm{w}}}{{G}_{\rm{c}}/{\delta }_{\rm{c}}}=1.89\times 1{0}^{-4}$, significant friction (*μ* = 0.35), and low viscosity *η*_reg_ = 0.05 s. Lastly, the meat-based food material is modeled with a high energy ratio $\frac{{G}_{\rm{c}}/{\delta }_{\rm{c}}}{G}=1.14\times 1{0}^{4}$, no adhesive shear stress ($\frac{{\tau }_{\rm{w}}}{{G}_{\rm{c}}/{\delta }_{\rm{c}}}=0$), no friction (*μ* = 0), and a high viscous resistance *η*_reg_ = 0.2 s. Regarding *η*_reg_, we note that although it is implemented as a viscous regularization (cf. Equation (31)), it also provides phenomenological insight into the complex cutting mechanisms of food materials with heterogeneous internal structures and the intermittent resistance as the blade advances (serration patterns in the experimental curve). The rulers on the right side in Fig. 3 provide a comparative overview of the parameters that govern the behavior of the cutting process. Furthermore, an animation of the simulated cutting processes is provided in Movie S4.

**Table 1:** Overview of the parameters of the model in relation to cutting mechanisms

Parameter	Description in relation to cutting mechanisms
Shear modulus (*G*)	Defines the stiffness of the bulk material and the energy stored during indentation. Influences how much strain energy is available to drive the propagation of a cut.
Poisson’s ratio (*ν*)	Governs the material’s lateral response under compression. Affects the bulk deformation around the blade during indentation and cutting.
Fracture toughness (*G*_c_)	Total energy required to create new cutting surfaces via cohesive failure. Determines the resistance to cutting initiation and propagation. Higher values lead to tougher, more cut-resistant behavior.
Characteristic opening displacement (*δ*_c_)	The maximum separation at which the cohesive traction reduces by a factor of 0.37. Sets the opening width needed to degrade cohesive forces–needs to be smaller than the cutting blade thickness.
Viscous damping regularization (*η*_reg_)	Introduces rate-dependence in the cohesive zone to improve numerical stability and mimic the effects observed during steady cutting of materials with internal heterogeneous structures like processed food. Allows for describing the damping effect in cutting initiation due to such heterogeneous structures.
Friction coefficient (*μ*)	Pressure-dependent Coulomb-type friction opposing relative sliding between blade and material, proportional to contact pressure. Significant during indentation and tight contact phases.
Critical shear stress for combined adhesion and wear (*τ*_W_)	Pressure-independent shear resistance due to adhesion (surface stickiness) and wear. Important for capturing the increasing cutting force observed in cohesive and sticky materials in the cutting regime due to contact with the cutting tool.

### Material behavior, fracture, and friction modulate the stability of the indentation-to-cutting transition

Using the computational model, we investigate the critical transition from indentation to cutting, revealing conditions under which the transition is abrupt or smooth. The model captures the role of dissipative forces in stabilizing the cutting process and explains experimentally observed differences across material systems.

The transition from indentation to cutting involves overcoming both the cohesive resistance at the initiation of the interface and energy dissipation through frictional forces. Once this energy threshold is crossed, two primary responses may occur: (i) an abrupt drop in cutting force, driven by the rapid release of stored elastic energy when dissipative mechanisms are weak, or (ii) a gradual transition, enabled by sufficient energy dissipation through frictional shear stresses at the tool-material interface and/or resistance due to damping contributions in the cohesive forces due to, e.g., heterogeneous internal structures of the material. These behaviors are evident in the experimental results shown in Fig. 2, clarifying how different dissipation pathways control the onset and nature of cutting. The gelatin hydrogel exhibits an unstable force drop characteristic of low dissipation, while the elastomer and meat-based material show smoother transitions, reflecting dominant damping.

For the gelatin hydrogel, the abrupt energy release and sharp drop in cutting force at the indentation-to-cutting transition are attributed to the high fracture energy *G*_c_ relative to the material stiffness *G*, combined with moderate tangential adhesive stresses that permit a low cutting force immediately after decohesion initiates (Fig. 3a.1). In contrast, the elastomer exhibits a smooth and stable transition, enabled by tangential dissipative forces from adhesion and wear, which absorb the energy released by the bulk during crack initiation (Fig. 3b.1). Coulomb friction further stabilizes the process by resisting sudden force drops. This stabilizing effect is confirmed in Fig. S7, where setting the friction coefficient from 0.35 to zero leads to a more abrupt force decrease, highlighting the role of friction in smoothing the cutting transition. Finally, the meat-based food material shows a delayed onset of cutting, consistent with a high *G*_c_/*G* ratio (Fig. 3c.1). In this case, large damping contributions in the cohesive forces at the cutting interface further resist decohesion, contributing to the postponed and smoother transition.

**Fig. 4:**
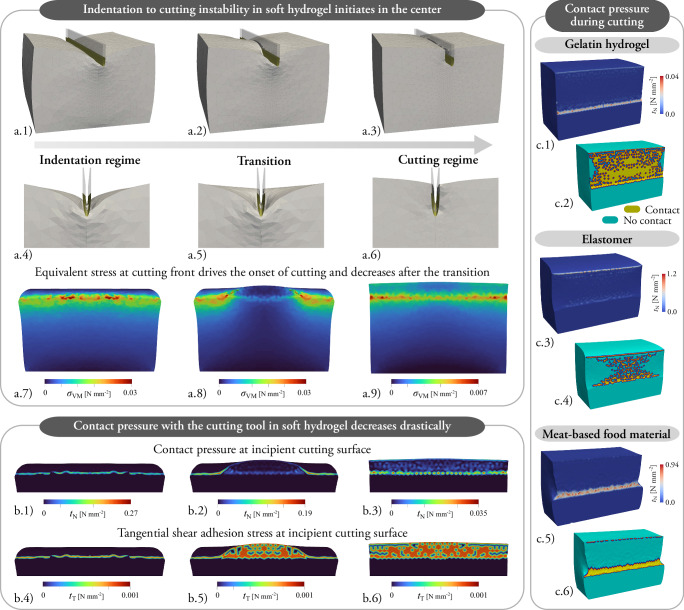
Constitutive behavior on the cutting surface investigated with the computational model: unstable indentation-to-cutting transition and contact pressure behind the crack front. **a.1–3** Unstable indentation-to-cutting transition in a gelatin hydrogel at an indentation of the cutting tool where the strain energy of the continuum suffices to overcome the cohesive energy to create a new cutting surface. The onset of cutting occurs at the center of the sample beneath the center of the cutting tool. **a.4-6** Detail of the transition with schematics of the cutting tool. **a.7–9** Equivalent Von Misses stress (*σ*_VM_) at the cutting surface driving the indentation-to-cutting transition. **b.1–3** Decrease in the contact pressure *t*_N_ in a gelatin hydrogel at the cutting surface. A normal stress prevents the interpenetration of material and the cutting tool along the transition. **b.4-6** Tangential contact stress *t*_T_ magnitude due to adhesion and wear along the transition. **c.1,3,5** Contact pressure behind the crack front at a stage after the cutting tool has advanced in the cutting regime (a displacement of the cutting tool of 13 mm) for the gelatin hydrogel, elastomer, and meat-based food material. The contact pressure in the cutting surface is negligible behind the cutting front---at the sides of the blade. **c.2,4,6** Binary field that represents the state of normal contact. Coulomb friction is zero in the areas where contact is inactive and minimal when contact occurs, but the contact pressure is negligible.

The onset of cutting at sufficiently large indentations initiates at the center of the sample—directly beneath the center of the blade—and propagates laterally as an instability toward the blade edges. Figure 4a.1–6 illustrates this mechanism for the gelatin hydrogel, the material exhibiting the most pronounced cohesive collapse. In this central region, the bulk material deforms under near plane-strain conditions, as it is laterally confined by the surrounding material. This constraint limits deformation along the longitudinal direction of the blade, activating a stiffer volumetric response against the penetrating tool. As a result, the stress concentration in the central region is higher, leading to premature failure. The equivalent von Mises stress, *σ*_VM_, along the indentation-to-cutting transition illustrates the mechanism. A peak, constant value of 0.03 N mm^−2^ relates to the onset of cutting in the central region, as depicted in Fig. 4a.7–9. Along the propagation of the cut in the longitudinal direction of the blade, *σ*_VM_ remains constant (Fig. 4a.8), and it drops to a value of 0.07 N mm^−2^ only at the end of the unstable transition corresponding to the beginning of the steady state cutting regime (see Fig. 4a.9).

As an alternative perspective, the maximum principal stress *σ*_1_ has often been employed in studies that use stress thresholds to predict the onset of cutting. To situate our findings within this context, we report the principal components of the Cauchy stress tensor in Fig. S8. At the top surface in contact with the cutting tool, the principal stress is compressive, whereas inside the material ahead of the process zone, it becomes tensile. This trend is consistent with the analysis of Goda and co-authors^58^. However, while those authors attribute cutting initiation to the free edges and subsequent propagation toward the mid-plane, our simulations indicate a slight preference for initiation within the mid-plane itself. Specifically, our model reveals slightly higher principal stresses in the mid-plane than at the lateral free surfaces (see Fig. S8b.1). It is important to emphasize that these findings lack experimental validation and relate to a particular boundary value problem: the cutting of a highly deformable cube-shaped hydrogel, subject to extreme deformations, contact interactions, and three-dimensional constraints. Consequently, these results may not directly apply to other cases. Overall, although stress-based failure criteria may suffice to address fracture initiation, we believe that our approach represents a paradigm shift from traditional stress-based predictions to process-based fracture modeling. To that end, we directly simulate the physical process of surface separation through cohesive zone modeling, which is based directly on energy release rates and fracture toughness rather than stress magnitudes and is irrespective of any stress-based failure criterion.

To rationalize the peak-drop behavior observed at the indentation-to-cutting transition in soft hydrogels, we additionally report a parametric study of the critical indentation depth and the indentation work at cutting onset as a function of the dimensionless parameter $\frac{{G}_{\rm{c}}/{\delta }_{\rm{c}}}{G}$. The shear modulus is varied while all other parameters remain fixed. As shown in Fig. S9a, the critical indentation depth (*d*_c_) increases with decreasing stiffness, ranging from 3.3 mm for *G* = 11 kPa to 6.8 mm for *G* = 4 kPa. A similar analysis for frictionless indentation is available in ref. ^12^. Figure S10b further depicts the external work performed by the blade during indentation up to cutting initiation (*W*_i_), which is slightly larger for small shear moduli. Although this trend may partly reflect numerical inaccuracies of the computational framework, it suggests that part of the work exerted by the tool is dissipated through tangential forces, an effect expected to intensify with larger non-linear indentation deformations in softer materials. Together with internal cohesive forces, friction forces at the interface between the material and the ellipsoidal blade tip resist the initiation of cutting by opposing the opening of the cutting surface, irrespective of whether these friction forces perform work during the indentation regime (corresponding to slip or stick conditions as defined in Equation (45)). Likewise, this difference is likely due to the effects of the boundary value problem and the intricate non-linear geometrical deformations related to it. Furthermore, additional simulations varying the dimensionless parameter $\frac{{\tau }_{\rm{w}}}{{G}_{\rm{c}}/{\delta }_{\rm{c}}}$ (i.e., the adhesive and wear maximum shear stress *τ*_w_) reveal that the cutting initiation point, defined by the indentation displacement and force, is only weakly affected by changes in this parameter. We note, however, that a stronger sensitivity is expected for larger blade sizes, owing to the increased contact area with the indenting tool.

During the unstable indentation-to-cutting transition in the gelatin hydrogel, the contact pressure with the cutting tool drops sharply. Figure 4b illustrates the evolution of the pressure during the indentation-to-cutting transition: the maximum value of 0.27 N mm^−2^ right before cutting onset drops progressively during the propagation of the cut to a steady value of 0.035 N mm^−2^. As a consequence, post onset, the only remaining tangential contact stresses are due to adhesion and wear, and not Coulomb friction, as illustrated in Fig. 4b.4-6. Coulomb friction effectively vanishes due to contact pressures dropping from maximum values of 0.27 N mm^−2^ to 0.035 N mm^−2^. In the cutting regime, the resulting tangential contact force becomes proportional to the area of material-tool contact, with the stress limited by the maximum adhesive and wear shear strength *τ*_w_. This value marks a transition from stick to slip conditions at the cutting interface.

### Adhesion, not Coulomb friction, controls tangential forces during cutting

Contrary to conventional assumptions, our results show that adhesive wear forces dominate tangential stresses during cutting, while Coulomb friction plays a negligible role due to low contact pressures. This finding reshapes the understanding of interfacial mechanics in soft cutting and guides improved material and tool design.

Not only immediately post cutting onset, but also throughout the cutting regime, tangential contact stresses due to Coulomb friction vanish as the cutting tool advances into the material. The continuum model enables detailed exploration of the normal contact pressure distribution at advanced stages of the cutting process. As shown in Fig. 4c.1, the gelatin hydrogel exhibits a peak contact pressure of 0.04 N mm^−2^ localized at the cutting front. Behind the front, the pressure drops sharply, with only marginal, localized residual values persisting along parts of the cutting surface (Fig. 4c.2). In the case of the elastomer, the highest contact pressure–reaching 1.2 N mm^−2^–appears along the upper edge of the cutting surface, while the regions trailing the crack front show minimal contact (Fig. 4c.3, 4). For the meat-based material, the peak contact pressure of 0.94 N mm^−2^ is similarly located at the crack front, with near-zero pressure along the remaining surface due to a prevailing non-contact condition (Fig. 4c.5, 6).

The continuum model for soft cutting reveals that friction does not contribute significantly to the tangential response observed experimentally, owing to the low contact pressures at the cutting surfaces. Instead, adhesive and wear-related contact forces fully account for the measured behavior. This interpretation is supported by the fact that the model is not sensitive to the value of the friction coefficient *μ* to reproduce the indentation-to-cutting transition of the gelatin hydrogel and meat-based materials, and by the observation of vanishing contact pressures during cutting. Even in the case of the elastomer, which has a non-zero *μ*, the contact pressure along the cutting interface drops to negligible values. As shown in Fig. S7, simulations with and without friction yield almost identical slopes in the cutting force-displacement curves, indicating that the tangential resistance arises exclusively from adhesion and wear. This conclusion is further supported by the contact pressure profiles in Fig. 4, which confirm that contact pressure along the cutting surfaces approaches zero during steady-state cutting.

Constraining the lateral expansion of the cube-shaped samples by restricting the motion of their side faces mimics the condition of a material embedded within a larger body, as would occur in alternative realistic cutting scenarios. This confinement may limit the ability of the material to deform freely in directions perpendicular to the cutting plane, thereby increasing the overall stiffness of the response against the penetrating tool. As a result, higher contact pressures may develop at the tool-material interface during cutting. We report additional cutting experiments on gelatin hydrogel samples with twice the original width, while maintaining the same cutting length, in Fig. S10. Contrary to the previous hypothesis, the results confirm width-independence of the Coulomb friction because increasing the lateral bulk volume does not significantly affect the cutting force response during the steady-state cutting regime, neither in terms of slope nor nominal force levels.

The blade size might, however, have a larger effect on the interfacial contact pressure. The elasto-cohesive length *l*_e_ = *G*_c_/*E*, for *E* the Young’s modulus, has been described as a function as the blade radius, setting the proximity between the blade and the crack tip and thus controlling the extent of contact pressure relative to the modulus, and in turn the balance between pressure-independent and pressure-dependent sliding resistance at the point above^17^. This point highlights a potential direction for further study.

## Discussion

Our results reveal that the mechanics of cutting in soft materials cannot be captured by classical fracture or friction theories alone. Instead, the transition from indentation to cutting is governed by a coupled interplay of constitutive response, material dissipation, contact-pressure-dependent friction, and contact-pressure-independent adhesion and wear. We showed that this transition can be either stable or unstable depending on material and interfacial parameters, that cutting initiation occurs centrally beneath the blade, and we described the role of adhesion in controlling the cutting force during the steady-state regime. Our model couples material and interfacial behavior on a comprehensive computational framework that substantiates, in the spirit of a virtual testbed, the understanding of adhesion- and friction-dominated cutting mechanisms, together with damping-mediated cutting due to material heterogeneity.

These findings provide a unifying framework for understanding why seemingly similar soft materials respond differently to the same cutting conditions. Beyond the specific systems studied here, the insights apply broadly to soft tissue manipulation, food processing, recycling of soft materials, and the design of flexible materials and soft robotic interfaces. Future extensions may incorporate data-driven identification of material parameters from observed cutting behavior, time-dependent contact evolution, viscoelastic material behavior, and cutting of poroelastic solids. Our findings can therefore be adapted to other contexts such as surgical dissection of soft anisotropic materials^59^ or food engineering^60,61^, including slice-push cutting mechanisms^62^, and also to materials processing in engineering contexts.

## Methods

### Materials

A gelatin hydrogel is produced by mixing animal-based gelatin, water, and glycerin. The solution contains 10% w/v of gelatin, with the liquid phase composed of 50% v/v water and 50% v/v glycerin. The solution was cast into a form and solidified at 10 °C. The samples were manufactured by mixing for 10 min, followed by storage in the fridge for 1 h.

Sylgard 184 (Dow Inc., Midland, Michigan, United States) was prepared mixing two raw phases in a 10:1—base to curing agent—volume mixing ratio. The crosslinked elastomer was cast into an open mold, cured at 90 °C for 2 h.

Processed meat-based food material consisting of 92% pork, complemented by drinking water and bacon. It contains—as disclaimed by the manufacturer—iodized salt (a mixture of table salt and potassium iodate), spice extracts such as fenugreek, chilli, ginger, cardamom, lovage, mace, and pepper, along with additional spices including chilli, coriander, onion, and marjoram. Dextrose is included as a sugar component, while diphosphates serve as stabilizers. Ascorbic acid acts as an antioxidant, and sodium nitrite is used as a preservative. The product is encased in pork intestine and finished with beechwood smoke.

The dimensions of the gelatin hydrogel and elastomer samples for cutting experiments are 30 mm (width *w*) × 30 mm (length in cutting direction) × 21 mm (height *h*). For the meat-based samples, the width is reduced to *w* = 25 mm due to the difficulty of shaping larger specimens and the length in the direction of the blade (cutting length) is kept equal.

The dimensions of the gelatin hydrogel, elastomer, and meat-based samples for tensile test experiments are 12 mm (width *w*) × 3 mm (thickness *t*) × 30 mm (length *l*).

### Cutting experiments

A universal tensile testing machine (Inspekt S 5 kN, Hegewald & Peschke, Nossen, Germany) was adapted to perform cutting experiments using a 0.7 mm-thick blade (wolfcraft, Kempenich, Germany), as shown in Fig. 1a and Fig. S1a. A custom-designed fixture was mounted on the upper crosshead to hold the blade and apply a quasi-static compressive displacement at a rate of 0.021 mm s^−1^, which renders an average strain rate of 0.001 s^−1^. The samples were positioned on a custom-designed support plate fixed to the lower crosshead of the testing machine. To ensure consistent cutting conditions, the blade was cleaned with lubricating oil after each experiment to remove any residual material or debris. All tests were conducted at room temperature, i.e., 23 °C.

### Digital image correlation

Pictures of the crack tip and surrounding area were taken during cutting experiments. A monochromatic CCD sensor (DCS 2.0, LIMESS Messtechnik & Software GmbH, Germany) with a resolution of 1024 × 768 and a lens with a focal range 50 mm and aperture 2.8 − 16 (2.8/50-0902 Xenoplan, Schneider Kreuznach, Bad Kreuznach, Germany) were used to capture the cracking pattern. Images were acquired at a rate of 1.01 Hz. The DIC postprocessing suite VIC-2D (Correlated Solutions Inc., Columbia, South Carolina) was used to compute the displacement and strain fields. A step size of 7 pixels and a subset of 27 pixels were used. In DIC, the step size is recommended to be less than one-third of the subset size. The strain field computed is obtained as the Lagrange strain tensor, ${\bf{E}}=\frac{1}{2}[{\bf{C}}-{\bf{I}}]$, with **C** the right Cauchy-Green tensor and **I** the second-order identity tensor.

The error fields between the experimental and numerical displacement fields shown in Fig. 3 in the main text are computed through a multi-step procedure. First, the numerical displacement field obtained from the FE simulation is interpolated onto a structured, equally spaced grid that matches the spatial resolution of the DIC experimental data. This ensures spatial correspondence between the two datasets over the lateral surface of the sample. The interpolated numerical data is then transformed into a matrix format consistent with the DIC output, where each entry corresponds to a pixel location (or subset, depending on the subset size) with appropriate scaling between millimeters and pixels. To enable direct comparison, the experimental displacement field is embedded into a matrix of the same dimensions as the numerical grid, using zero-padding along the margins. The error field is then calculated as the pointwise difference between the experimental and numerical displacement fields, and visualized in the deformed configuration, using the experimentally measured horizontal and vertical DIC displacements to represent the deformation.

### Tensile experiments

An universal tensile machine (Inspekt S 5 kN, Hegewald & Peschke, Nossen, Germany) was used to perform tensile tests on pre-cut rectangular samples—initial notch of 2.4 mm—with a quasi-static tensile loading rate of 0.03 mm s^−1^, which renders an average strain rates of 0.001 s^−1^. The clamps on the machine were actuated with air pressure. The force-displacement data were stored during the deformation of the samples. All tests were conducted at room temperature, i.e., 23 °C.

### Unified computational model for soft cutting: finite strains, cohesive fracture, and contact

We propose a unified framework that combines a discontinuous spatial discretization at a cohesive interface—referred to as the cutting cohesive surface—with a contact formulation with an ideally rigid cutting tool. The model incorporates tangential friction governed by Coulomb’s law—proportional to the contact pressure up to the point of slipping—as well as adhesion, modeled as a constant stress activated upon contact.

Consequently, the cutting resistance experienced by the tool arises from four main contributions:

i. the fracture toughness or cohesive strength of the material,
ii. tangential frictional stress at the interface due to relative motion between the tool and the material, i.e., Coulomb friction,
iii. adhesive debonding stress, and
iv. shear stress associated with wear—that is, surface damage occurring in the sample.

We note that the decomposition of the total frictional resistance into a pressure-dependent component (ii) and pressure-independent contributions (iii and iv) has been discussed in the context of puncture mechanics in ref.^16^. In the present work, the pressure-independent contribution is taken to encompass both adhesive and wear effects.

### Kinematic framework

Let us consider a solid undergoing inhomogeneous deformation induced by a displacement discontinuity, as illustrated in Fig. S1b. This strong discontinuity defines the cutting plane. Initially, the body is represented by a single connected domain, ${{\mathcal{B}}}_{0}$, which subsequently separates into two disjoint parts. In the material configuration, the cutting cohesive surface, i.e., the plane of separation, is denoted by *S*_0_, and it partitions the solid into two distinct halves

1 $${{\mathcal{B}}}_{0}={{\mathcal{B}}}_{0}^{-}\cup {{\mathcal{B}}}_{0}^{+}.$$

The two halves lie on the associated plus and minus sides of the cut surface

2 $${S}_{0}={S}_{0}^{-}\cup {S}_{0}^{+}.$$

Simultaneously, let the cutting surface consist of contact and cohesive parts, as illustrated in Fig. S1c,

3 $${S}_{0}^{\pm }={S}_{\rm{contact,0}}^{\pm }\cup {S}_{\rm{cohesive,0}}^{\pm }.$$

The deformation of the medium is formulated in a finite strain framework. The deformation ${\boldsymbol{\varphi }}({\bf{X}})$ maps the positions in the material configuration ${\bf{X}}\in {{\mathcal{B}}}_{0}$ to the positions in the spatial configuration ${\bf{x}}\in {\mathcal{B}}$ *via*

4 $${\bf{x}}={\boldsymbol{\varphi }}({\bf{X}})={\bf{u}}({\bf{X}})+{\bf{X}}.$$

Here, **u** denotes the displacement field. In the spatial configuration, the two cutting surfaces are denoted by *S*^+^ and *S*^−^, and the corresponding deformed regions of the body are ${{\mathcal{B}}}^{+}$ and ${{\mathcal{B}}}^{-}$. A surface traction will be defined later to enforce cohesion and to allow for eventual separation resulting from cutting the solid. This separation is characterized by the displacement jump across the cutting surface

5 $${[\![} {\bf{u}}{]\!]}={{\bf{u}}}^{+}-{{\bf{u}}}^{-}.$$

Let us now define the material unit normal vector **N** as the outward normal to the boundary of the solid domain. Similarly, let **N**^±^ denote the unit normals on the positive and negative sides of the cohesive surface *S*_0_, each pointing outward from the respective subdomains ${{\mathcal{B}}}_{0}^{\pm }$ as illustrated in Fig. S1b. In the spatial configuration, let **n** represent the unit normal to the outer boundary of the deformed body ${\mathcal{B}}$, and let **n**^±^ be the corresponding unit normals on either side of the discontinuous cutting interface *S*.

Consistently, the normal and tangential components of the displacement jump read

6 $${[\![}{\bf{u}}{]\!]}_{{\rm{n}}}={[[\![}{\bf{u}}{]\!]}\cdot \bar{{\bf{n}}}]\bar{{\bf{n}}}\quad {\text{and}}\quad{[\![}{\bf{u}}{]\!]}_{{\rm{t}}}=[{\bf{I}}-\bar{{\bf{n}}}\otimes \bar{{\bf{n}}}]\cdot {\bf{u}},$$

with the average spatial unit normal vector computed as $\overline{{\bf{n}}}=[{{\bf{n}}}^{-}-{{\bf{n}}}^{+}]/| | {{\bf{n}}}^{-}-{{\bf{n}}}^{+}| |$, for ${\bf{n}^{\pm}}={[{\bf{F}}^{\rm{-T}}\cdot {\bf{N}}]}^{\pm}/| | {\bf{F}}^{\rm{-T}}\cdot {\bf{N}}|{|}^{\pm}$. The negative sign in front of **n**^+^ is necessary to reverse the orientation of the vector, as the face normal in each subdomain is defined to point outward from its respective cell. Without reversing the orientation of one of the vectors, their normal contributions would cancel out due to opposing directions.

The average deformation mapping for the deformed cutting surface *S* further characterizes the deformation of the cohesive surface, allowing to identify a unique deformed configuration of *S*. It reads

7 $$\{{\bf{u}}\}=\frac{1}{2}[{{\bf{u}}}^{+}+{{\bf{u}}}^{-}].$$

The Dirichlet and Neumann boundaries of the solid are denoted, respectively, by ∂_D_ and ∂_N_

8 $${\partial }_{{\rm{D}}}{{\mathcal{B}}}_{0}={\partial }_{{\rm{D}}}{{\mathcal{B}}}_{0}^{+}\,\cup \,{\partial }_{{\rm{D}}}{{\mathcal{B}}}_{0}^{-}\quad {\text{and}}\quad {\partial }_{{\rm{N}}}{{\mathcal{B}}}_{0}={\partial }_{{\rm{N}}}{{\mathcal{B}}}_{0}^{+}\,\cup \,{\partial }_{{\rm{N}}}{{\mathcal{B}}}_{0}^{-}.$$

Note that ${\partial }_{\rm{D}}{{\mathcal{B}}}_{0}$ and ${\partial }_{\rm{N}}{{\mathcal{B}}}_{0}$ do not include the cutting surface *S*_0_.

The deformation gradient is defined as

9 $${\bf{F}}={\nabla }_{0}{\bf{u}}+{\bf{I}}$$

with **I** the second-order identity tensor and ∇_0_ the gradient operator in the material configuration. Following the multiplicative isochoric-volumetric decomposition into volumetric (**F**_vol_) and isochoric ($\overline{{\bf{F}}}$) parts,

10 $${\bf{F}}={{\bf{F}}}_{\rm{vol}}\cdot \overline{{\bf{F}}},$$

with

11 $${{\bf{F}}}_{{\rm{v}}{\rm{o}}{\rm{l}}}={[\det {\bf{F}}]}^{1/3}{\bf{I}}\quad {\text{and}}\quad \bar{{\bf{F}}}={[\det {\bf{F}}]}^{-1/3}{\bf{F}},$$

and $\det {\bf{F}}$ the determinant of **F**.

### Strong and weak forms

The strong formulation of the problem reads

12 $${{\boldsymbol{\nabla }}}_{0}\cdot {\bf{P}}+{{\bf{b}}}_{0}={\bf{0}},\quad {\text{in}}\,{{\mathcal{B}}}_{0}^{\pm },$$

13 $${\bf{P}}\cdot {\bf{N}}={{\bf{t}}}^{{\rm{p}}/{\rm{c}}},\quad {\text{on}}\,{\partial }_{{\rm{N}}}{{\mathcal{B}}}_{0}^{\pm },$$

14 $${\bf{u}}={{\bf{u}}}^{{\rm{p}}}, \quad {\text{on}}\,{\partial }_{{\rm{D}}}{{\mathcal{B}}}_{0}^{\pm },$$

15 $${[{\bf{P}}\cdot {\bf{N}}]}^{\pm }={{\bf{t}}}^{{\rm{c}},\pm },\quad {\text{on}}\,{S}_{{\rm{c}}{\rm{o}}{\rm{n}}{\rm{t}}{\rm{a}}{\rm{c}}{\rm{t}},0}^{\pm },$$

16 $${{\bf{P}}}^{+}\cdot {{\bf{N}}}^{+}+{{\bf{P}}}^{-}\cdot {{\bf{N}}}^{-}={\bf{0}},\quad {\text{on}}\,{S}_{{\rm{c}}{\rm{o}}{\rm{h}}{\rm{e}}{\rm{s}}{\rm{i}}{\rm{v}}{\rm{e}},0}^{\pm },$$

with **t**^p/c^ a generic traction that is either prescribed (superscript “p”) or due to contact with the cutting tool (superscript “c”) and **u**^p^ a prescribed displacement.

The weak formulation of the field equations in Equations (12)–(16) is obtained by multiplying with a test function *δ***u** and integrating by parts,

17 $$\begin{array}{rcl} & & -{\int }_{{{\mathcal{B}}}_{0}}{\bf{P}}:{\nabla }_{0}\delta {\bf{u}}\,{\rm{d}}V+{\int }_{{\partial }_{{\rm{N}}}{{\mathcal{B}}}_{0}}{{\bf{t}}}^{{\rm{p}}/{\rm{c}}}\cdot \delta {\bf{u}}\,{\rm{d}}A+\mathop{\sum }\limits_{\pm }{\int }_{{S}_{{\rm{c}}{\rm{o}}{\rm{n}}{\rm{t}}{\rm{a}}{\rm{c}}{\rm{t}},0}^{\pm }}{{\bf{t}}}^{{\rm{c}}}\cdot \delta {\bf{u}}\,{\rm{d}}A\\ & & +{\int }_{{S}_{{\rm{c}}{\rm{o}}{\rm{h}}{\rm{e}}{\rm{s}}{\rm{i}}{\rm{v}}{\rm{e}},0}^{+}}{{\bf{P}}}^{+}\cdot {{\bf{N}}}^{+}\cdot \delta {{\bf{u}}}^{+}{\rm{d}}A+{\int }_{{S}_{{\rm{c}}{\rm{o}}{\rm{h}}{\rm{e}}{\rm{s}}{\rm{i}}{\rm{v}}{\rm{e}},0}^{-}}{{\bf{P}}}^{-}\cdot {{\bf{N}}}^{-}\cdot \delta {{\bf{u}}}^{-}{\rm{d}}A=0.\end{array}$$

For simplicity, and given that the soft cutting model neglects body forces, the corresponding term has been omitted from Equation (17).

A further manipulation of the weak form can be done to express it in terms of the discontinuous operators—jump and average—across the cutting surface. Taking **N**^−^ as reference normal vector at the cutting interface, the traction on the positive side can be expressed as **P**^+^ ⋅ **N**^+^ = − **P**^+^ ⋅ **N**^−^ and the re-formulated weak form

18 $$\begin{array}{lll} & & -{\int }_{{{\mathcal{B}}}_{0}}{\bf{P}}:{\nabla }_{0}\delta {\bf{u}}\,{\rm{d}}V+{\int }_{{\partial }_{{\rm{N}}}{{\mathcal{B}}}_{0}}{{\bf{t}}}^{{\rm{p}}/{\rm{c}}}\cdot \delta {\bf{u}}\,{\rm{d}}A+\mathop{\sum }\limits_{\pm }{\int }_{{S}_{{\rm{c}}{\rm{o}}{\rm{n}}{\rm{t}}{\rm{a}}{\rm{c}}{\rm{t}},0}^{\pm }}{{\bf{t}}}^{{\rm{c}}}\cdot \delta {\bf{u}}\,{\rm{d}}A\\ & & -{\int }_{{S}_{{\rm{c}}{\rm{o}}{\rm{h}}{\rm{e}}{\rm{s}}{\rm{i}}{\rm{v}}{\rm{e}},0}}{[\![}{\bf{P}}\cdot \delta {\bf{u}}{]\!]}\cdot {{\bf{N}}}^{-}{\rm{d}}A=0.\end{array}$$

The split of the jump of the product as ⟦**P** ⋅ *δ***u**⟧ = {**P**} ⋅ ⟦*δ***u**⟧ + ⟦**P**⟧ ⋅ {*δ***u**} renders

19 $$\begin{array}{lll} & & {\int }_{{{\mathcal{B}}}_{0}}{\bf{P}}:{\nabla }_{0}\delta {\bf{u}}\,{\rm{d}}V+{\int }_{{S}_{{\rm{c}}{\rm{o}}{\rm{h}}{\rm{e}}{\rm{s}}{\rm{i}}{\rm{v}}{\rm{e}},0}}\{{\bf{P}}\}\cdot {{\bf{N}}}^{-}\cdot {[\![}\delta {\bf{u}}{]\!]}\,{\rm{d}}A+\\& &{\int }_{{S}_{{\rm{c}}{\rm{o}}{\rm{h}}{\rm{e}}{\rm{s}}{\rm{i}}{\rm{v}}{\rm{e}},0}}{[\![}{\bf{P}}{]\!]}\cdot {{\bf{N}}}^{-}\cdot \{\delta {\bf{u}}\}\,{\rm{d}}A=\\ & & {\int }_{{\partial }_{{\rm{N}}}{{\mathcal{B}}}_{0}}{{\bf{t}}}^{{\rm{p}}/{\rm{c}}}\cdot \delta {\bf{u}}\,{\rm{d}}A+\mathop{\sum }\limits_{\pm }{\int }_{{S}_{{\rm{c}}{\rm{o}}{\rm{n}}{\rm{t}}{\rm{a}}{\rm{c}}{\rm{t}},0}^{\pm }}{{\bf{t}}}^{{\rm{c}}}\cdot \delta {\bf{u}}\,{\rm{d}}A,\end{array}$$

where ${[\![}\delta {\bf{u}}{]\!]}=\delta [{{\bf{u}}}^{+}-{{\bf{u}}}^{-}]$ denotes the jump operator across the cutting surface.

Eventually, the balance of tractions in the cohesive part of the cutting surface, *S*_cohesive,0_, (Equation (16)) cancels the integral ${\int }_{{S}_{\rm{cohesive,0}}}[\![{\bf{P}}]\!]\cdot {{\bf{N}}}^{+}\cdot \{\delta {\bf{u}}\}\,{\rm{d}}A$ in Equation (19), yielding the final most convenient variant of the weak form

20 $$\begin{array}{rcl} & & {\int }_{{{\mathcal{B}}}_{0}}{\bf{P}}:{\nabla }_{0}\delta {\bf{u}}\,{\rm{d}}V+{\int }_{{S}_{{\rm{c}}{\rm{o}}{\rm{h}}{\rm{e}}{\rm{s}}{\rm{i}}{\rm{v}}{\rm{e}},0}}\{{\bf{P}}\}\cdot {{\bf{N}}}^{-}\cdot {[\![}\delta {\bf{u}}{]\!]}\,{\rm{d}}A=\\& &{\int }_{{\partial }_{{\rm{N}}}{{\mathcal{B}}}_{0}}{{\bf{t}}}^{{\rm{p}}/{\rm{c}}}\cdot \delta {\bf{u}}\,{\rm{d}}A+\mathop{\sum }\limits_{\pm }{\int }_{{S}_{{\rm{c}}{\rm{o}}{\rm{n}}{\rm{t}}{\rm{a}}{\rm{c}}{\rm{t}},0}^{\pm }}{{\bf{t}}}^{{\rm{c}}}\cdot \delta {\bf{u}}\,{\rm{d}}A.\end{array}$$

The Galerkin approximation of the trial and test functions consists of piecewise functions in the Sobolev subspace, specifically ${\bf{u}}\in {{\mathcal{H}}}^{1}$ and $\delta {\bf{u}}\in {{\mathcal{H}}}^{1}$. These functions are continuous within the subdomains ${{\mathcal{B}}}_{0}^{+}$ and ${{\mathcal{B}}}_{0}^{-}$, but exhibit a discontinuity across the cohesive surface *S*_0_. We note that an alternative implementation could rely on a fully discontinuous Galerkin finite-dimensional approximation of the fields^56^, which enables crack propagation along any finite element interface. While this offers a more general and flexible framework, in the context of cutting, the crack path is, in principle, constrained to follow the cutting surface.

The duality, or work-conjugacy, relations between stress and deformation measures are also evident in Equation (20). Similar to conventional solids, the Piola stress tensor **P** performs work on the deformation gradient **F** throughout the bulk of the material, while the traction {**P**} ⋅ **N**^−^ contributes to the work by acting on the displacement jumps across the discontinuous cutting interface. The opening displacement ⟦**u**⟧ serves as a measure of deformation, while the tractions act as the corresponding conjugate stress measure.

### Failure: cohesive zone model

In the context of cohesive debonding of the interface, the term ${\int }_{{S}_{\rm{cohesive,0}}}\{{\bf{P}}\}\cdot {{\bf{N}}}^{-}\cdot {[\![}\delta {\bf{u}}{]\!]}\,{\rm{d}}A$ in the weak form (Equation (20)) represents the virtual work done by the cohesive traction force across the cutting interface and can be rewritten in terms of the cohesive traction (c.f., e.g., ^63,64^),

21 $${\int }_{{S}_{\rm{cohesive,0}}}\{{\bf{P}}\}\cdot {{\bf{N}}}^{-}\cdot {[\![}\delta {\bf{u}}{]\!]}\,{\rm{d}}A:={\int }_{{S}_{\rm{cohesive,0}}}{\bf{T}}({[\![}{\bf{u}}{]\!]})\cdot {[\![}\delta {\bf{u}}{]\!]}\,{\rm{d}}A.$$

The damage process, which involves the formation of new crack surfaces, is modeled using virtual cohesive elements. This is achieved by progressively reducing the cohesive traction as the separation between the bulk elements in a potential crack extension zone increases. Failure occurs when the cohesive traction vanishes upon exceeding a critical separation threshold. A traction-separation law governs the constitutive behavior of decohesion, which can be defined in terms of the cohesive free energy density per unit undeformed area, *W*_s_, over *S*_0_^55,65^, along with the cohesive law ${\bf{T}}({[\![}{\bf{u}}{]\!]})$,

22 $${\bf{T}}({[\![}{\bf{u}}{]\!]})=\frac{\partial {W}_{\rm{s}}}{\partial {[\![}{\bf{u}}{]\!]}}.$$

In turn, the cohesive law can be expressed in terms of the normal and tangential components—representing the resistance to normal opening and sliding, respectively—as

23 $${\bf{T}}=\frac{\partial {W}_{\rm{s}}}{\partial {[\![}{\bf{u}}{{]\!]}}_{\rm{n}}}{\bf{n}}+\frac{\partial {W}_{\rm{s}}}{\partial {[\![}{\bf{u}}{{]\!]}}_{\rm{t}}}\frac{{[\![}{\bf{u}}{{]\!]}}_{\rm{t}}}{\parallel {[\![}{\bf{u}}{{]\!]}}_{\rm{t}}\parallel }.$$

To simplify the formulation of mixed-mode cohesive laws, the effective opening displacement^66^ is defined as

24 $$\delta ({[\![}{\bf{u}}{]\!]})=\left\{\begin{array}{ll}\sqrt{{[\![{\bf{u}}]\!]}_{{\rm{n}}}\cdot {[\![{\bf{u}}]\!]}_{{\rm{n}}}+{\gamma }_{{\rm{t}}}\,{[\![{\bf{u}}]\!]}_{{\rm{t}}}\cdot {[\![{\bf{u}}]\!]}_{{\rm{t}}}}, & \text{if}\,{[\![{\bf{u}}]\!]}_{{\rm{n}}}\ge 0,\\ \sqrt{{\gamma }_{{\rm{t}}}\,{[\![{\bf{u}}]\!]}_{{\rm{t}}}\cdot {[\![{\bf{u}}]\!]}_{{\rm{t}}}}, & \text{if}\,{[\![{\bf{u}}]\!]}_{{\rm{n}}} < 0,\end{array}\right.$$

where *γ*_t_ is a parameter that weights the normal and sliding opening displacements. This definition allows to prevent damage when the positive and negative sides of the cutting surface interpenetrate, i.e., when ⟦**u**⟧ _n_ < 0.

Letting *W*_s_ be a function of the effective opening, Equation (23) can then be reformulated as

25 $${\bf{T}}=\frac{1}{\delta }\frac{\partial {W}_{\rm{s}}(\delta )}{\partial \delta }[{[\![}{\bf{u}}{{]\!]}}_{\rm{n}}+{\gamma }_{\rm{t}}^{2}{[\![}{\bf{u}}{{]\!]}}_{\rm{t}}],$$

where $t(\delta )=\partial {W}_{\rm{s}}(\delta )/\partial \delta$ defines the effective traction.

Let us adopt a potential based on Smith and Ferrante’s law, i.e., one of the seminal simplified representations to calculate the cohesive energy in solids (cf.^55,67^),

26 $${W}_{{\rm{s}}}=e{\sigma }_{{\rm{c}}}{\delta }_{c}\left[1-\left[1+\frac{\delta }{{\delta }_{{\rm{c}}}}\right]{e}^{-\delta /{\delta }_{{\rm{c}}}}\right],$$

with *e* ≈ 2.71828, *σ*_c_ the maximum cohesive normal traction and *δ*_c_ a characteristic opening displacement. Note that the prefactor *e* in Equation (26) normalizes the potential so that the minimum energy is exactly − *σ*_c_*δ*_*c*_ at *δ* = *δ*_c_. It is a scaling factor introduced for convenience and consistency.

In addition, we introduce a rate-dependent regularization of the cohesive free energy density allows to regularize the opening of the cutting surface at the transition from indentation to cutting regimes. The dependence on the pseudo-time is introduced with the term $\frac{1}{2}{\eta }_{{\rm{r}}{\rm{e}}{\rm{g}}}[\![\mathop{{\bf{u}}}\limits^{.}]\!]\cdot [\![\mathop{{\bf{u}}}\limits^{.} ]\!]$, where the time rate of the jump of the displacement field can be approximated as $[\![\mathop{{\bf{u}}}\limits^{.}]\!]\approx [[\![{\bf{u}}{]\!]}_{i}-[\![{\bf{u}}{]\!]}_{i-1}]/\Delta t$, with Δ*t* the pseudo-time increment. The regularization term is then added up to the cohesive energy as $W={W}_{{\rm{s}}}+\frac{1}{2}{\eta }_{{\rm{r}}{\rm{e}}{\rm{g}}}[\![\mathop{{\bf{u}}}\limits^{.}]\!]\cdot [\![\mathop{{\bf{u}}}\limits^{.}]\!]$. This will produce additional cohesive forces proportional to $[\![\mathop{{\bf{u}}}\limits^{.}]\!]$ that can damp the abrupt opening of the cutting surface at the onset of the cutting regime. We note that viscous cohesive forces according to our approach may not vanish after complete decohesion if $[\![\mathop{{\bf{u}}}\limits^{.}]\!]\,\ne \, {\bf{0}}$. This does not occur in our computations as both flanks of the cutting surface remain separated exactly by the cutting tool and tangential motion is minimal. A more general formulation of viscous cohesive forces may imply degrading such forces with the damage state variable *d*.

As a consequence, the cohesive law reads

27 $${\bf{T}}([\![{\bf{u}}]\!])=e\frac{{\sigma }_{{\rm{c}}}}{{\delta }_{\rm{c}}}[{{\rm{e}}}^{-\delta /{\delta }_{{\rm{c}}}}[[\![{\bf{u}}{]\!]}_{{\rm{n}}}+{\gamma }_{{\rm{t}}}^{2}[\![{\bf{u}}{]\!]}_{{\rm{t}}}]+{\eta }_{{\rm{r}}{\rm{e}}{\rm{g}}}[\![\mathop{{\bf{u}}}\limits^{.}]\!]].$$

For convenience and to enforce damage irreversibility, let *d* represent a damage state variable

28 $$d=\mathop{\max }\limits_{t\in [0,t]}(1-{e}^{-\delta /{\delta }_{\rm{c}}}).$$

As a consequence of the state variable, an unloading event, which results in $\mathop{\delta }\limits^{.} < 0$, leads to a linear dependence between the cohesive traction and the effective opening. Note that the choice of *d* as internal variable is equivalent to setting the maximum attained effective opening displacement ${\delta }_{\max }$ as internal variable.

Fracture criteria in cohesive zone modeling are based on an energy balance for crack nucleation and propagation. Crack initiation occurs when the energy available to separate the cohesive zone is sufficient. In non-linear elastic materials, a computation of the *J*-integral establishes the following link between the critical energy release rate *G*_c_ for crack propagation and the cohesive law (see^55^),

29 $${G}_{{\rm{c}}}={\int }_{0}^{\infty }T(\delta ){\rm{d}}\delta \qquad {\text{with}}\qquad T(\delta )=e\frac{{\sigma }_{{\rm{c}}}}{{\delta }_{\rm{c}}}{{\rm{e}}}^{-\delta /{\delta }_{{\rm{c}}}}.$$

Note that equation (29) denotes the limit of the cohesive free energy density *W*_s_ as the opening displacement approaches infinity.

For the case of Smith and Ferrante’s law in Equation (26), the fracture toughness reads

30 $${G}_{\rm{c}}=e{\sigma }_{\rm{c}}{\delta }_{\rm{c}}\quad \to \quad e\frac{{\sigma }_{\rm{c}}}{{\delta }_{\rm{c}}}=\frac{{G}_{\rm{c}}}{{\delta }_{\rm{c}}^{2}}.$$

Note that the energy release rate *G* is equivalent to the *J*-integral and the cohesive energy *Γ*_0_ in the absence of energy dissipation around the crack tip. This equivalence holds only at crack initiation. Once the crack propagates, elastic unloading becomes inevitable, and the energy consumed during cracking is less than the *J*-integral. As a result, it cannot be directly quantified from the global energy balance^68^.

In the sequel, the cohesive law can be re-written in terms of the damage variable and fracture toughness,

31 $${\bf{T}}([\![{\bf{u}}]\!])=\frac{{G}_{{\rm{c}}}}{{\delta }_{{\rm{c}}}^{2}}[[1-d][[\![{\bf{u}}{]\!]}_{{\rm{n}}}+{\gamma }_{{\rm{t}}}^{2}[\![{\bf{u}}{]\!]}_{{\rm{t}}}]+{\eta }_{{\rm{r}}{\rm{e}}{\rm{g}}}[\![\mathop{{\bf{u}}}\limits^{.}]\!]],$$

with *d* the damage variable defined in Equation (28).

The fracture parameters *G*_c_ and *δ*_c_ are both constitutive parameters that must be calibrated simultaneously to ensure that the fracture toughness *Γ*_0_ aligns with experimental observations. For the sake of convenience, we calibrate the ratio *G*_c_/*δ*_c_ rather than both individual parameters. Since *δ*_c_ also appears in Equation (28), its value needs to be prescribed independently. Here, it is important to note that *δ*_c_ must be sufficiently small to guarantee complete decohesion between the two halves, ${{\mathcal{B}}}_{0}^{\pm }$, across the width of the cutting tool. For convenience, we assume that *δ*_c_ ≪ *a*, where *a* will be defined in the following sections as half the width of the cutting tool. This ensures that the contact pressure between the solid and the tool is only due to the deformation of the bulk material and not to residual cohesive forces—not fully degraded—between both sides of the cohesive surface.

As a practical criterion, we set *δ*_c_ = 2*a*/10, i.e., ten times smaller than the width of the cutting tool, taking the value of 0.07 mm in this work. This is sufficiently small to guarantee full decohesion for a separation equal to the width of the cutting tool. Equation (31) defines the cohesive law as a function of the opening displacement. The reader will observe that $| | {\bf{T}}| | ({[\![}{\bf{u}}{]\!]})$ for a normal opening of ⟦**u**⟧ _n_ = 2*a* = 0.7 mm, i.e., the width of the blade as defined later, is nearly zero.

### Contact with the cutting tool on the cutting surface

The formulation for contact between the material and cutting tool at the cutting surface is incorporated as contact traction forces on the + and − sides of the contact part of the cutting surface, ${S}_{\rm{contact,0}}^{\pm }$. To this end, the related term is retrieved from the weak form (Equation (20)) and reformulated in terms of normal contact traction forces to prevent interpenetration (${{\bf{t}}}_{\rm{N}}^{\rm{c}}$) and tangential contact traction forces for friction with the cutting tool (${{\bf{t}}}_{\rm{T}}^{\rm{c}}$), i.e.,

32 $$\begin{array}{rcl} & & \mathop{\sum }\limits_{\pm }{\int }_{{S}_{{\rm{c}}{\rm{o}}{\rm{n}}{\rm{t}}{\rm{a}}{\rm{c}}{\rm{t}},0}^{\pm }}{{\bf{t}}}^{{\rm{c}}}\cdot \delta {\bf{u}}\,{\rm{d}}A=\\ & & \underbrace{{\int }_{{S}_{{\rm{c}}{\rm{o}}{\rm{n}}{\rm{t}}{\rm{a}}{\rm{c}}{\rm{t}},0}^{+}}{{\bf{t}}}_{{\rm{N}}}^{{\rm{c}},+}\cdot \delta {{\bf{u}}}^{+}\,{\rm{d}}A+{\int }_{{S}_{{\rm{c}}{\rm{o}}{\rm{n}}{\rm{t}}{\rm{a}}{\rm{c}}{\rm{t}},0}^{-}}{{\bf{t}}}_{{\rm{N}}}^{{\rm{c}},-}\cdot \delta {{\bf{u}}}^{-}\,{\rm{d}}A}_{{\rm{N}}{\rm{o}}{\rm{r}}{\rm{m}}{\rm{a}}{\rm{l}}\,{\rm{c}}{\rm{o}}{\rm{n}}{\rm{t}}{\rm{a}}{\rm{c}}{\rm{t}}:\,{\rm{O}}{\rm{b}}{\rm{s}}{\rm{t}}{\rm{a}}{\rm{c}}{\rm{l}}{\rm{e}}\,{\rm{p}}{\rm{r}}{\rm{o}}{\rm{b}}{\rm{l}}{\rm{e}}{\rm{m}}}+\\&&\underbrace{{\int }_{{S}_{{\rm{c}}{\rm{o}}{\rm{n}}{\rm{t}}{\rm{a}}{\rm{c}}{\rm{t}},0}^{+}}{{\bf{t}}}_{{\rm{T}}}^{{\rm{c}},+}\cdot \delta {{\bf{u}}}^{+}\,{\rm{d}}A+{\int }_{{S}_{{\rm{c}}{\rm{o}}{\rm{n}}{\rm{t}}{\rm{a}}{\rm{c}}{\rm{t}},0}^{-}}{{\bf{t}}}_{{\rm{T}}}^{{\rm{c}},-}\cdot \delta {{\bf{u}}}^{-}\,{\rm{d}}A}_{{\rm{T}}{\rm{a}}{\rm{n}}{\rm{g}}{\rm{e}}{\rm{n}}{\rm{t}}{\rm{i}}{\rm{a}}{\rm{l}}\,{\rm{c}}{\rm{o}}{\rm{n}}{\rm{t}}{\rm{a}}{\rm{c}}{\rm{t}}:\,{\rm{F}}{\rm{r}}{\rm{i}}{\rm{c}}{\rm{t}}{\rm{i}}{\rm{o}}{\rm{n}}\,\& \,{\rm{a}}{\rm{d}}{\rm{h}}{\rm{e}}{\rm{s}}{\rm{i}}{\rm{o}}{\rm{n}}}.\end{array}$$

Note that we consider contact and friction separate phenomena to be modeled independently of the cohesive law.

### Contact with the cutting tool on the external boundary during indentation

Furthermore, contact during the initial indentation on the outer boundary is modeled with the traction force boundary integral in the weak form (Equation (20)) according to

33 $$\int_{{\partial _{\rm{N}}}{{\mathcal{B}}_0}} {{{\bf{t}}^{{\rm{p}}/{\rm{c}}}} \cdot \delta {\bf{u}}{\rm{dA}} = \int_{{\partial _{\rm{N}}}{{\mathcal{B}}_0}} {{{\bf{t}}^{\rm{p}}} \cdot \delta {\bf{u}}{\rm{dA}}} + \underbrace {\int_{{\partial _{\rm{N}}}{{\mathcal{B}}_0}} {{\bf{t}}_{\rm{N}}^{\rm{c}} \cdot } \delta {\bf{u}}{\rm{dA}}}_{{\rm{Normal}}\,{\rm{contact}}:\,{\rm{Obstacle}}\,{\rm{problem}}} + \underbrace {\int_{{\partial _{\rm{N}}}{{\mathcal{B}}_0}} {{\bf{t}}_{\rm{T}}^{\rm{c}} \cdot } \delta {\bf{u}}{\rm{dA}}.}_{{\rm{Tangential}}\,{\rm{contact}}:\,{\rm{Coulomb}}\,{\rm{friction}}\,\&\, {\rm{adhesion}}}}$$

Note that the term including **t**^p^ refers to any additionally prescribed traction force on the external boundary.

### Normal contact with cutting tool: obstacle problem and augmented Lagrangian formulation

A slave surface is defined as the boundary of the deformable body, denoted by $\partial {{\mathcal{B}}}_{0}$, whereas the master surface is modeled as a smooth two-dimensional surface $\Omega \subset {{\mathbb{R}}}^{3}$, parameterized by coordinates ***ξ*** ∈ *Ω*, representing an ideally rigid cutting tool.

The gap function—also referred to as distance or penetration function^69,70^—is defined as

34 $${g}_{\rm{N}}=\mathop{\min }\limits_{{\bf{x}}\subset \partial {\mathcal{B}}}| | {\bf{x}}-{\boldsymbol{\xi }}| | .$$

Subsequently, the following states of the contact area can be defined as

$$\begin{array}{cl}{g}_{{\rm{N}}} < 0 & {\text{no}}\,{\text{contact}},\\ {g}_{{\rm{N}}}=0 & {\text{perfect}}\,{\text{contact}},\\ {g}_{{\rm{N}}} > 0 & {\text{penetration}}.\end{array}$$

The requirement that the deformable solid must not penetrate the cutting tool gives rise to a contact constraint, which is mathematically expressed through the Karush-Kuhn-Tucker (KKT) conditions

35 $$\it{g}_{\rm{N}}\le 0,\quad \it{p}_{\rm{N}}\ge 0,\quad \it{g}_{\rm{N}}\,\it{p}_{\rm{N}}=0,$$

where *p*_N_ denotes the contact pressure that arises as a reaction force associated with the constraint *g*_N_ = 0 in the case of active contact.

We define the master surface representing the cutting tool, as described in Section 4.2, to consist of an ellipsoidal tip and a constant-thickness rectangular segment of the blade (see Fig. S1c). Let *Ω* = *Ω*_e_ ∪ *Ω*_r_ denote the surface of the cutting tool, where the subscript “e” refers to the ellipsoidal part and “r” to the rectangular upper segment. Accordingly, an ad hoc definition for the gap function is given by

36 $${g}_{{\rm{N}}}=\left\{\begin{array}{l}\begin{array}{ll}{g}_{{\rm{N}},{\rm{e}}}, & {\text{if}}\,\min | | {\bf{x}}-{\boldsymbol{\xi }}| | \to {\boldsymbol{\xi }}\in {\Omega }_{{\rm{e}}},\end{array}\\ \begin{array}{cc}{g}_{{\rm{N}},{\rm{r}}}, & {\text{if}}\,\min | | {\bf{x}}-{\boldsymbol{\xi }}| | \to {\boldsymbol{\xi }}\in {\Omega }_{{\rm{r}}}.\end{array}\end{array}\right.$$

The geometric definition of the ellipsoidal tip and rectangular segment can be expressed *via* the following formalism:

37 $${g}_{\rm{N,ellip.}}=1-{[{\bf{x}}-{\bf{c}}]}^{\rm{T}}\cdot {{\bf{A}}}_{1}\cdot [{\bf{x}}-{\bf{c}}],$$

38 $${g}_{\rm{N,rect.}}=1-{[{\bf{x}}-{\bf{c}}]}^{\rm{T}}\cdot {{\bf{A}}}_{2}\cdot [{\bf{x}}-{\bf{c}}],$$

where ${\bf{c}}={[{c}_{1},{c}_{2},{c}_{3}]}^{\rm{T}}$ is the position vector of the center of the ellipse, and **A**_1_ is a symmetric matrix representing the shape and orientation of the ellipse, modeling the tip of the cutting blade. For an ellipse lying in the *xy*-plane, we have ${{\bf{A}}}_{1}={\rm{diag}}({a}^{-2},{b}^{-2},0)$, where *a* and *b* are the lengths of the semi-axes of the ellipse. For the blade used in the experiments (see Section 4.2), we set *a* = 0.35 mm. On the other hand, **A**_2_ is a matrix that represents the width and orientation of a rectangular upper band—of infinite vertical length—modeling the constant-thickness part of the cutting blade. Letting *a* represent the small semi-axis of the ellipse (which corresponds to the thickness of the cutting tool), we define ${{\bf{A}}}_{2}={\rm{diag}}({a}^{-2},0,0)$.

To implement the obstacle problem we employ an augmented Lagrangian framework. The framework regularizes the normal contact problem defined in Equation (35) as proposed in the works of Simo et al.^57^. Consequently, the normal traction force ${{\bf{t}}}_{\rm{N}}^{\rm{c}}$ in Equations (32) and (33) can be expressed in terms of a Lagrange multiplier *λ*_N_ as

39 $${{\bf{t}}}_{\rm{N}}^{\rm{c}}=-\langle {\lambda }_{\rm{N}}^{\pm }+\frac{\epsilon_{\rm{N}}}{\it{h}}\it{g}_{\rm{N}}({{\bf{u}}}^{\pm })\rangle {{\bf{n}}}^{\pm },$$

with the spatial normal vector computed as **n** = **F**^−T^ ⋅ **N**/∣∣**F**^−T^ ⋅ **N**∣∣, *ϵ*_N_ = 1 the penalty parameter for the augmentation scheme, *h* the mesh size calculated as the diameter of the cell (elements in the FE mesh), and 〈•〉 Macaulay brackets. Note that the penalty is regularized with the mesh size, since in the limit *h* → 0 a fixed penalty parameter would cause the penalty contribution in the weak form to vanish, rendering the constraint ineffective.

Note that the obstacle problem constraint can be satisfied even if *ϵ*_N_ is undersized through repeated application of the augmentation procedure, preventing at the same time ill-conditioning of the resulting matrix problem.

The multiplier in Equation (39) is then augmented in successive iterations according to an Uzawa update scheme, i.e.,

40 $${\lambda }_{\rm{N},\it{k}\rm{+1}}^{\pm }=\langle {\lambda }_{\rm{N},\it{k}}^{\pm }+\frac{{\epsilon }_{\rm{N}}}{\it{h}}\it{g}_{\rm{N}}({{\bf{u}}}^{\pm })\rangle .$$

Note that the basic Uzawa iteration^71^ consists of two steps: i) minimize the weak form to find **u** and reduce the violation of the constraint (gap function), keeping the Lagrange multiplier *λ*_N_ fixed; ii) update the multiplier to penalize the constraint violation, proportionally to how much the violation remains. After successive iterations, Equation (40) drives the normal gap *g*_N_ to zero, effectively enforcing the contact constraint and transferring the corresponding contact traction to the Lagrange multiplier *λ*_N_.

### Tangential contact with cutting tool: Coulomb friction, adhesion and penalty regularization

Tangential contact forces accounting for friction, adhesion, and wear are introduced through ${{\bf{t}}}_{\rm{T}}^{\rm{c}}$ in Equations (32) and (33). We distinguish between two primary mechanisms that give rise to shear stress: friction (pressure-dependent) and adhesion/wear (pressure-independent). Friction is modeled via Coulomb’s law and leads to slipping when the tangential contact traction ${{\bf{t}}}_{\rm{T}}^{\rm{c}}$ exceeds the product of a friction coefficient *μ* and the normal pressure. Adhesion, on the other hand, arises from the bonding strength between the material and the cutting tool, as well as from the resistance to wear (i.e., surface damage) of the material^17^. The combined adhesive and wear shear strength can reach a maximum value denoted by *τ*_W_.

Depending on the magnitude of the normal pressure, the contact forces exerted by the cutting tool may be dominated either by friction—under high contact pressures—or by adhesion and wear resistance—under low contact pressures. In practical terms, the parameter *τ*_W_ serves to characterize the combined adhesive and wear shear strength and governs the frictional response when the normal contact pressure is small.

The Karush-Kuhn-Tucker conditions for Coulomb friction read

41 $$\mathop{{\bf{u}}}\limits^{.}-{\mathbb{[}}\mathop{{\bf{c}}}\limits^{.}{\mathbb{]}}=\rho \frac{\partial \Phi }{\partial {{\bf{t}}}_{{\rm{T}}}^{{\rm{c}}}},$$

42 $$\rho \ge 0,$$

43 $$\rho \,\Phi =0,$$

with *ρ* a Lagrange multiplier and **c** the position vector of the cutting tool, hence $\mathop{{\bf{c}}}\limits^{.}$ is its rate of change and $\mathop{{\bf{u}}}\limits^{.}-\mathop{{\bf{c}}}\limits^{.}$ is the relative motion of the contact surface of the solid and the cutting tool along the successive iterations. Note that the relative motion $\mathop{{\bf{u}}}\limits^{.}-\mathop{{\bf{c}}}\limits^{.}$ is tangential upon friction due to the interpenetration contact condition in Equation (35). Note that in our formulation the center of the cutting tool, **c**, is defined as the center of an ideally ellipsoidal cutting tool. Nonetheless, the reader may note that only the relative motion $\mathop{{\bf{c}}}\limits^{.}$ of the cutting tool in the cutting direction is required.

The slip criterion, *Φ*, is defined as

44 $$\Phi :={t}_{\rm{T}}^{\rm{c}}-[\mu \, {t}_{\rm{N}}^{\rm{c}}+{\tau }_{\rm{W}}\, \Theta ({t}_{\rm{N}}^{\rm{c}})]\le 0,$$

and it establishes whether slip or perfect stick occur according to

45 $$\Phi =\left\{\begin{array}{l}\begin{array}{ll} < 0 & \to \ perfect\,stick\,{\text{condition}},\end{array}\\ \begin{array}{ll}=0 & \to \ slip\,{\text{condition}}.\end{array}\end{array}\right.$$

Further, $\Theta ({t}_{\rm{N},\it{n}\rm{+1}}^{\rm{c}})$ is an activation function to activate tangential contact due to adhesion and wear constant forces such that

46 $$\Theta ({t}_{{\rm{N}}})=\left\{\begin{array}{l}\begin{array}{ll}1 & {\text{if}}\ {t}_{{\rm{N}}}^{{\rm{c}}} > 0,\end{array}\\ \begin{array}{cc}0 & {\text{otherwise}}.\end{array}\end{array}\right.$$

In the absence of normal contact, i.e., when ${t}_{\rm{N}}^{\rm{c}}=0$, the contribution of *τ*_W_ in the friction potential *Φ* (Equation (44))—which accounts for tangential dissipative forces due to adhesion and wear—is deactivated, resulting in ${t}_{\rm{T}}^{\rm{c}}=0$. Once normal contact is established, i.e., ${t}_{\rm{N}}^{\rm{c}} > 0$, the switching function $\Theta ({t}_{\rm{N},\it{n}{\rm{+1}}}^{\rm{c}})$ transitions from zero to one, thereby activating the contribution of *τ*_W_ and extending the flow potential *Φ* accordingly.

The simplest approach to enforce the friction constraint involves transforming the constrained minimization problem into an unconstrained one by applying a penalty regularization to the frictional obstacle problem:

47 $${{\bf{t}}}_{\rm{T},\it{n}{\rm{+1}}}^{\rm{c,trial}}={{\bf{t}}}_{\rm{T},\it{n}}^{\rm{c}}+\frac{{\epsilon }_{\rm{T}}}{\it{h}}[{{\bf{u}}}_{\it{n}{\rm{+1}}}-{{\bf{u}}}_{\it{n}}{\rm{}}+{{\bf{c}}}_{\it{n}{\rm{+1}}}-{{\bf{c}}}_{\it{n}}{\rm{]}},$$

48 $${\Phi }_{\it{n}{\rm{+1}}}^{\rm{trial}}={t}_{\rm{T},\it{n}{\rm{+1}}}^{\rm{c,trial}}-[\mu \, {t}_{\rm{N},\it{n}{\rm{+1}}}^{\rm{c}}+{\tau }_{W} \, \Theta ({t}_{\rm{N},\it{n}{\rm{+1}}}^{\rm{c}})],$$

with *ϵ*_T_ = 1 × 10^8^ the penalty parameter for Coulomb friction.

The return mapping is completed with the following expression for the traction force to be inserted in Equations (32) and (33)

49 $${{\bf{t}}}_{\rm{T},\it{n}\rm{+1}}^{\rm{c}}={{\bf{t}}}_{\rm{T},\it{n}{\rm{+1}}}^{\rm{c,trial}}-\Delta \zeta \frac{{{\bf{t}}}_{\rm{T},\it{n}{\rm{+1}}}^{\rm{c,trial}}}{\parallel {{\bf{t}}}_{{\rm{T}},\it{n}{\rm{+1}}}^{\rm{c,trial}}\parallel },$$

with Δ*ζ* the traction correction in the direction in the trial tangential force. The correction vanishes for perfect stick condition and it actuates under the slip condition according to

50 $$\Delta \zeta =\left\{\begin{array}{l}\begin{array}{ll}0, & {\text{if}}\,{\Phi }_{n+1}^{\mathrm{trial}}\le 0,\\ {\Phi }_{n+1}^{\mathrm{trial}}, & {\text{if}}\,{\Phi }_{n+1}^{\mathrm{trial}} > 0.\end{array}\end{array}\right.$$

### Bulk material behavior: hyperelastic constitutive model

Only the definition of a constitutive model for the bulk material remains to be defined. Let the energy density per undeformed volume *Ψ* be composed of isochoric and volumetric contributions, following the decoupled representation

51 $$\Psi ({\bf{F}})={\Psi }_{\rm{iso}}(\overline{{\bf{F}}})+{\Psi }_{\rm{vol}}(\det {\bf{F}}).$$

The isochoric contribution to the total energy density in Equation (51) is defined according to the neo-Hookean model as

52 $${\Psi }_{\rm{iso}}(\overline{{\bf{F}}})=\frac{\it{G}}{2}\left[{\bf{I}}:\left[{\overline{{\bf{F}}}}^{\rm{T}}\cdot \overline{{\bf{F}}}\right]-3\right],$$

with *G* the shear modulus. The calibrated values of *G* are detailed in Table 2.

**Table 2:** Calibration of parameters of the in silico model

Material	Bulk material		Cohesive fracture		Friction & adhesion		Viscous
	*G* [MPa]	*ν* [–]	*δ*_c_ [mm]	*G*_c_/*δ*_c_ [N m^−2^]	*μ* [−]	*τ*_W_ [N m^−2^]	*η*_reg_ [s]
Gelatin hydrogel	7.5 × 10^−3^	0.49	0.07	75.7	0	0.0008	0.05
Elastomer	120 × 10^−3^	0.49	0.07	214.3	0.35	0.04	0.05
Meat-based food material	25 × 10^−3^	0.49	0.07	285.7	0	0	0.2

The parameters of the unified model for soft cutting are direct descriptors of the cutting mechanisms, namely the bulk, cohesive fracture, and interfacial mechanisms.

For the volumetric contribution, we use a relation dependent on the bulk modulus that is adequate to recover the nearly incompressible behavior of elastomers, i.e.,

53 $${\Psi }_{{\rm{v}}{\rm{o}}{\rm{l}}}(J)=\frac{\kappa }{2}{[J-1]}^{2},\quad {\text{with}}\quad \kappa =\frac{2G[1+\nu ]}{3[1-2\nu ]},$$

for bulk modulus *κ* with Poisson ratio *ν* set to 0.49 to recover nearly incompressibility.

We note that the constitutive response to volumetric deformations plays a crucial role in determining the cutting force (see, e.g., ref. ^72^). Notably, even slight reductions in Poisson’s ratio within the nearly incompressible regime can lead to significant changes in both the onset of cutting and the magnitude of the cutting force. In this work, we implement a compressible model with Poisson’s ratio close to the incompressible limit and an object of future works may be the implementation of a mixed formulation for incompressibility, e.g., with Lagrange multipliers.

An additional direction for future work is the extension of the model to incorporate viscoelastic or poroelastic behaviors, achieved by introducing non-equilibrium contributions to the free energy and potentially viscous cohesive laws.

Lastly, the Piola stress tensor can be derived from the energy density as the addition of isochoric and volumetric contributions. The isochoric contribution reads

54 $${{\bf{P}}}_{\rm{iso}}=\dfrac{\partial {\Psi }_{\rm{iso}}(\overline{{\bf{F}}})}{\partial {\bf{F}}}=\it{J}^{\rm{-1/3}}{\mathbb{K}}:\dfrac{\partial {\Psi }_{\rm{iso}}(\overline{{\bf{F}}})}{\partial \overline{{\bf{F}}}}=\it{G}\,{J}^{\rm{-1/3}}{\mathbb{K}}:\overline{{\bf{F}}},$$

with the fourth-order mixed-variant projection tensor ${\mathbb{K}}={\mathbb{I}}-\frac{1}{3}{{\bf{F}}}^{\rm{-T}}\otimes {\bf{F}}$, and the volumetric contribution

55 $$\begin{array}{r}{{\bf{P}}}_{\rm{vol}}=\dfrac{\partial {\Psi }_{\rm{vol}}({\bf{F}})}{\partial {\bf{F}}}=\it{J}\dfrac{\partial {\rm{\Psi} }_{\rm{vol}}({\bf{F}})}{\partial J}{{\bf{F}}}^{\rm{-T}}=\kappa \left[\it{J}^{\rm{2}}-\it{J}\right]{{\bf{F}}}^{-T}.\end{array}$$

### Numerical Implementation

The weak form in Equation (20) is implemented in the latest version of the open-source finite element platform FEniCS^73–75^: FEniCSx v0.9.0. The Galerkin discretization of each of the two subdomains next to the cutting surface is done with linear Lagrange polynomial basis functions. The implementation of the mixed continuous Galerkin framework—continuous subdomains (${{\mathcal{B}}}_{0}^{\pm }$) and discontinuous cohesive interface (*S*_0_)—submeshes were utilized as described in ^76^. A FE mesh with tetrahedral elements is used to discretize the computation domain with finite-dimensional approximation of functions. The mesh for the gelatin hydrogel and elastomer samples (width of 30 mm, cutting length of 30 mm, and height of 21 mm) has 47,801 elements, and the mesh for the meat-based food material (width of 25 mm, cutting length of 30 mm, and height of 21 mm) has 45,456 elements. The displacement at the bottom surface in contact with the plate is fixed to zero in all directions via Dirichlet boundary conditions. This mimics the nearly-ideal adhesion of the samples in the experiments.

The high non-linearity of the variational problem requires staggered numerical resolution strategies:

- The decohesion of the material ahead of the cutting tool modeled by means of the traction-separation law in Equation (31) is addressed with a fixed-point resolution strategy. For a fixed time step, the displacement field **u**_*i*_ at a time step *i* is solved for a damage field fixed to a previously known value *d*_*i*−1_^76^. Then, the damage field is updated to *d*_*i*_ using **u**_*i*_. The calculation is repeated iteratively until the error between iterations is under a tolerance.
- The stepwise activation of friction due to adhesion and wear (Equation (46)) is evaluated at a previously known value of the contact pressure, i.e., $\Theta ({t}_{\rm{N},\it{i}\rm{-1}})$. Once the displacement field **u**_*i*_ is determined, *Θ* is updated to $\Theta ({t}_{\rm{N},\it{i}})$. The iteration loop is the same as the aforementioned one for the numerical update of damage on the cohesive interface.
- Further, to prevent spurious decohesion due to oscillations of *t*_N_, which might be non-positive spontaneously on the sides of the cutting tool, $\Theta ({t}_{\rm{N},\it{n}\rm{+1}})$ is kept active once contact between the cutting surface *S*_0_ and the cutting tool has occurred.

The transition from the initial indentation regime to the cutting regime is a highly nonlinear process that necessitates sufficiently small time steps to ensure convergence. To balance accuracy and efficiency, we employ an adaptive load-stepping strategy that dynamically adjusts the time step based on the evolution of the damage variable. Specifically, when the increment in damage between successive steps exceeds a threshold of Δ*d*_*i*→*i*+1_ = 0.1, the time step is reduced by a factor of 10, down to a minimum of Δ*t* = 0.0001. Conversely, if the damage grows more moderately, the time step is increased by a factor of 5, up to a maximum of Δ*t* = 0.2. The total pseudo-time is set to *t*_end_ = 21, which matches the maximum indentation depth of the cutting tool. This ensures that the indentation process is controlled directly via the simulation pseudo-time, with both quantities defined to be equal.

The viscous regularization of Equation (31) works hand-in-hand with the adaptive load-stepping solver. High values of the pseudo-time rate of the jump displacement $[\![\mathop{{\bf{u}}}\limits^{.}]\!]$ lead to high-damping cohesive forces. However, the reader may note that the pseudo-time plays a role in the approximation $[\![\mathop{{\bf{u}}}\limits^{.}]\!]\approx [[\![{\bf{u}}{]\!]}_{i}-[\![{\bf{u}}{]\!]}_{i-1}]/\Delta t$. Should the simulation halt the displacement of the cutting tool with Δ*t* → 0, and provided that the transition from indentation to cutting regimes is an instability, the regularization forces would tend to infinity. As a consequence, the minimum value of the pseudo-time step needs to remain greater than zero.

### Overview of parameters of the model: physical meaning and calibration

The parameters of the model are direct descriptors of the physics in the cutting process. Table 1 describes them in relation to the cutting mechanism. In regard to the parameter *η*_reg_ and its use in this work to rationalize damped cutting arising from internal material heterogeneity, the authors suggest that future research may explore the development of soft-fracture cohesive laws and/or bulk constitutive models that explicitly capture the decohesion in heterogeneous structures.

The parameters of the computational model are calibrated to reproduce the experimental cutting curves and summarized in Table 2.

## Supplementary information

Supplementary Information

Movie S1

Movie S2

Movie S3

Movie S4

## Data Availability

The data generated during the study are available via Zenodo at 10.5281/zenodo.15873109.
