# Supplementary material for: Cutting soft materials: how material differences shape the response
Source: NPJ Comput Mater. 2026 Jan 6;12(1):15. doi: 10.1038/s41524-025-01869-y (PMC12789027; doi:10.1038/s41524-025-01869-y)
Supplement: Supplementary file 1 — Supplementary Information [file 41524_2025_1869_MOESM1_ESM.pdf]

npj | computational materials

Supporting Information for

Cutting soft materials: how material differences shape the response

Miguel Angel Moreno-Mateos and Paul Steinmann

Correspondence to: Miguel Angel Moreno Mateos.
E-mail: miguel.moreno@fau.de

This PDF file includes:

Supporting text
Figs. S1 to S10
Legends for Movies S1 to S4
SI References

Other supporting materials for this manuscript include the following:

Movies S1 to S4

Supporting Information Text

1. Overview of experimental and computational testbed for cutting of soft solids

Figure S1 provides an overview of the experimental and virtual setup for cutting of soft materials.

2. Additional results for cutting of gelatin hydrogels with alternative compositions

Two additional gelatin solutions were fabricated by mixing gelatin powder with a liquid mixture of water and glycerin in a different proportion: a solution containing 13.33 %w/v gelatin, with the liquid phase composed of 66.67 %v/v water and 33.33 %v/v glycerin; and a solution containing 10 %w/v gelatin, with the liquid phase composed of 100 %v/v water. The original one in the main manuscript is more hyperelastic than the two variants in this appendix, which are more brittle due to the lower content of glycerin. The results for the respective hydrogels are shown in Figures S2 and S3.

3. Results for cutting of additional food materials

Additional results for cutting experiments on food materials: cheese, tofu, and marshmallow. The results are shown in Figure S4. The food materials are classified according to the three cutting mechanisms described in the main text: adhesion-dominated, adhesion- and friction-dominated, and viscous-dominated.

Emmental cheese exhibits an abrupt transition from indentation to cutting, followed by a second peak in the cutting force. Subsequently, the cutting force increases due to tangential adhesion with the cutting tool. This behavior may be influenced by the geometry of the cutting tool and the fat content of the cheese. Overall, the cutting response resembles that of the gelatin hydrogel discussed in the main text.

Tofu shows a smooth indentation-to-cutting transition, resembling the viscous cutting behavior observed in the meat-based food material presented earlier. After the onset of cutting, the cutting force decreases progressively rather than abruptly. As previously argued for meat-based samples, this may be due to the heterogeneous structure of the material and its moisture content. Notably, the cutting force in tofu decreases over time, in contrast to the relatively stable force observed in the meat-based sample. This difference may point to the role of water diffusion in tofu, which could be explored in future studies.

Marshmallow displays a smooth indentation-to-cutting transition with noticeable remanent deformation in the bulk material. Although adhesion may occur, as suggested by the increasing cutting force after the onset of cutting, the cutting behavior does not clearly fit into any of the three proposed categories. Future work may investigate the constitutive behavior of this sugar-based material and its underlying damage mechanisms.

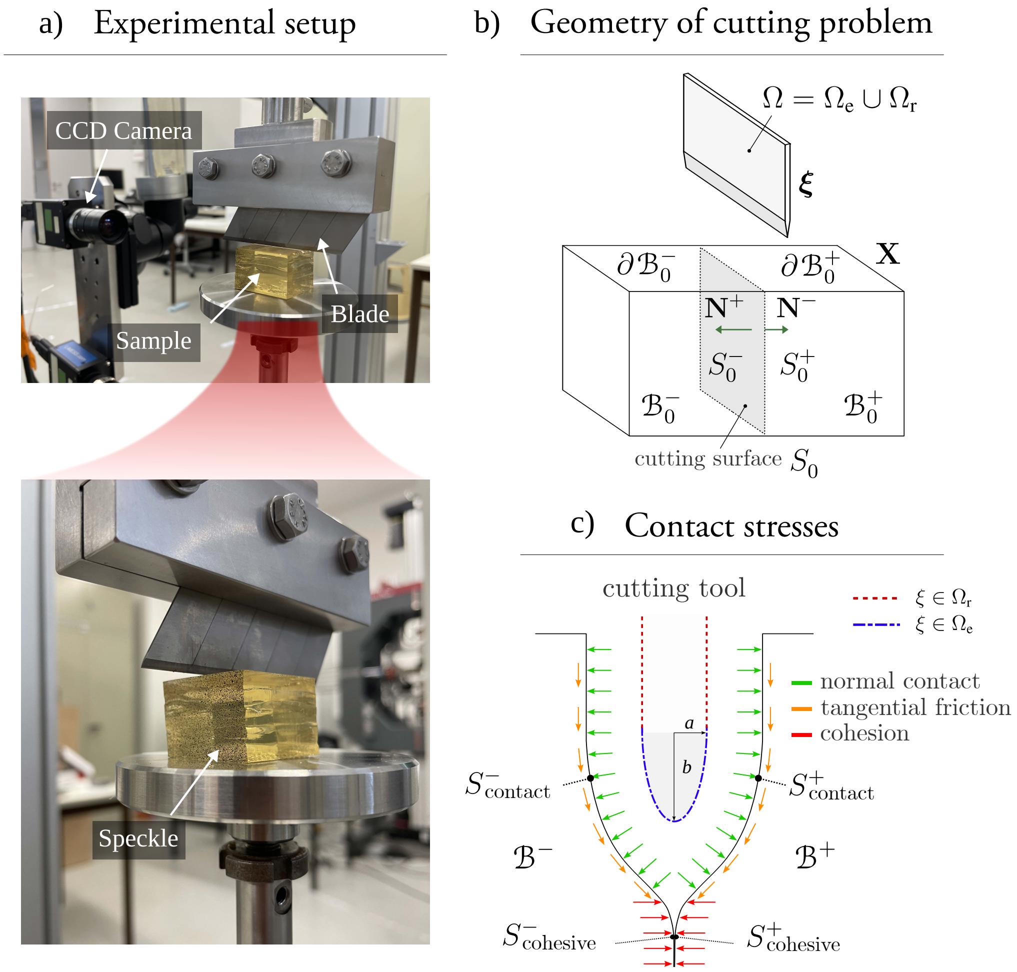

Figure S1. Overview of the experimental and virtual setup for cutting of soft materials. (a) Experimental setup used for cutting experiments. (b) Schematics of the solid and cutting tool with nomenclature of the medium, its boundaries, and cohesive surface. (c) Schematics of the balance of interfacial forces—contact-normal and tangential—forces on the contact boundary with the cutting tool, and cohesive forces on the crack front. The deformable medium is split into two regions through normal and tangential contact interactions with the cutting tool, which is modeled as a smooth mathematical surface. The tool comprises two segments: an upper region, Ω_r, representing a rectangular section of infinite vertical extent, and a lower region, Ω_e, forming an ellipsoidal tip. At the interface between the two halves of the medium, cohesive tractions are applied to resist separation, as governed by a cohesive law that depends explicitly on the displacement jump across the interface.

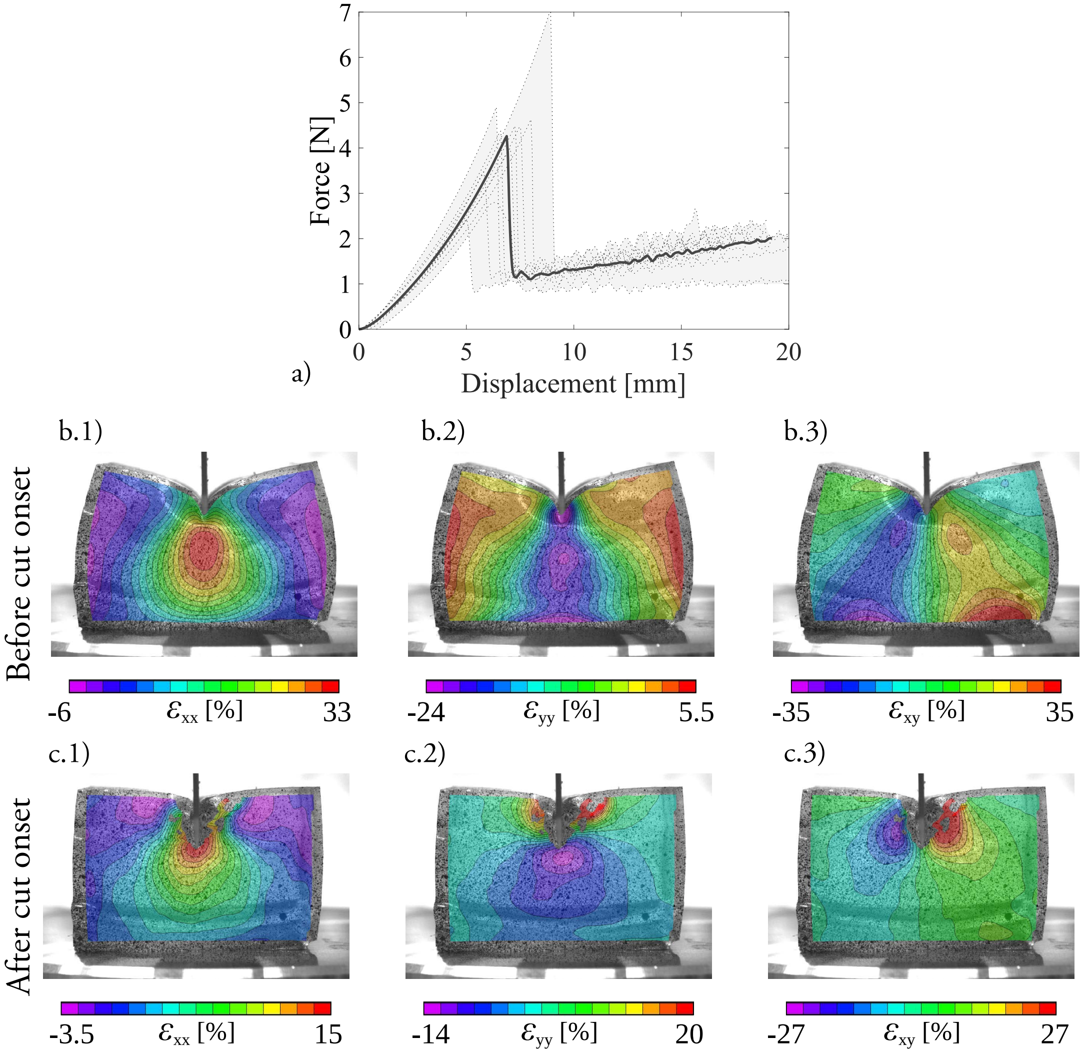

Figure S2. Results for experimental cutting tests on a first variant of the gelatin hydrogel. A first variant is a solution containing $13.33\,\%\mathrm{w/v}$ gelatin, with the liquid phase composed of $66.67\,\%\mathrm{v/v}$ water and $33.33\,\%\mathrm{v/v}$ glycerin. Before cutting onset, i.e., at the end of the initial indentation, and after cutting onset. (a) Force-indentation curves for ten repetitions under the same test conditions. (b) Engineering strain fields (defined according to the main text, Methods) for one of the experimental repetitions. The data corresponds to one of the experimental array for equal test conditions. Note that a test with a force–displacement curve close to the average one is selected.

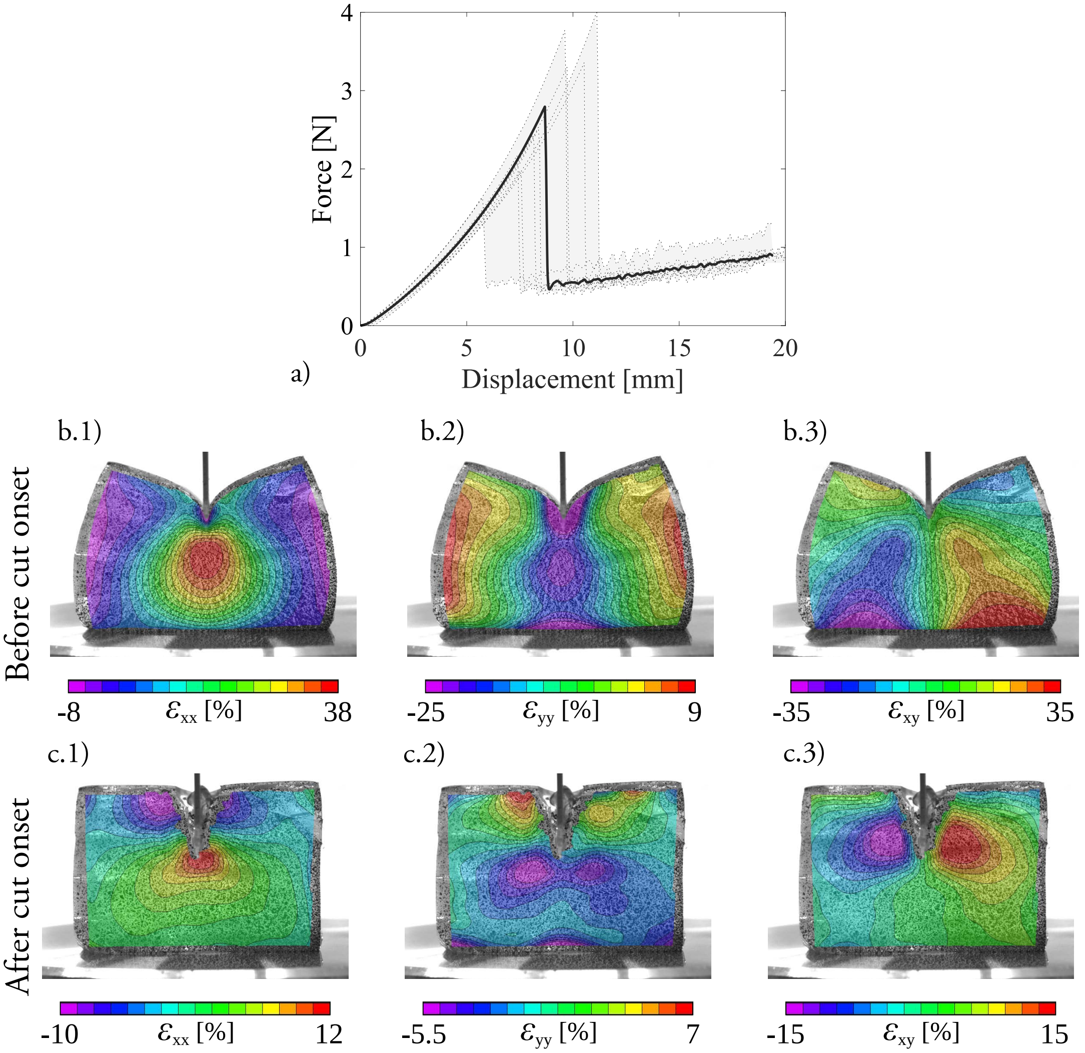

Figure S3. Results for experimental cutting tests on a second variant of the gelatin hydrogel. A second variant is a solution containing $10\,\%\mathrm{w/v}$ gelatin, with the liquid phase composed of $100\,\%\mathrm{v/v}$ water. Before cutting onset, i.e., at the end of the initial indentation, and after cutting onset. (a) Force-indentation curves for ten repetitions under the same test conditions. (b) Engineering strain fields (defined according to the main text, Methods) for one of the experimental repetitions. The data corresponds to one of the experimental array for equal test conditions. Note that a test with a force–displacement curve close to the average one is selected.

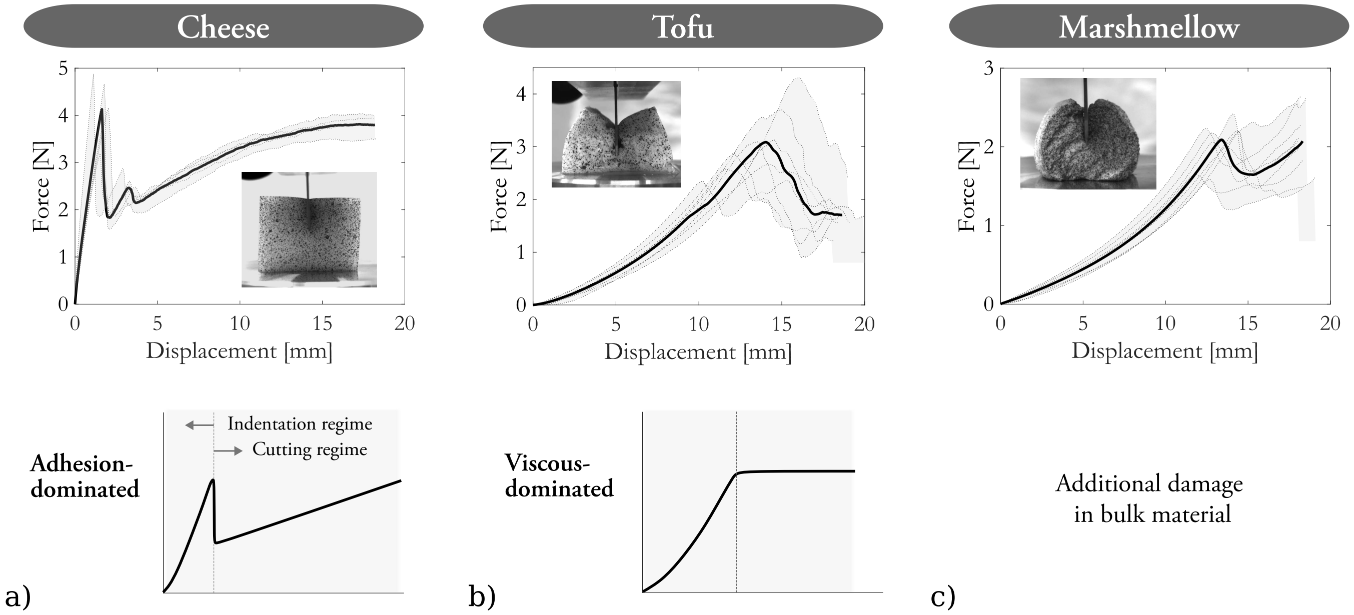

Figure S4. Results for cutting experiments on additional food materials. Cutting force versus displacement curves for emmental cheese, tofu, and marshmellow. The dimensions for cheese samples are $30\,\mathrm{mm}$ (width w) $\times$ $30\,\mathrm{mm}$ (length in cutting direction) $\times$ $21\,\mathrm{mm}$ (height h); for tofu samples, $30\,\mathrm{mm}$ (width w) $\times$ $30\,\mathrm{mm}$ (length in cutting direction) $\times$ $21\,\mathrm{mm}$ (height h); for marshmellow samples, $18\,\mathrm{mm}$ (width w) $\times$ $18\,\mathrm{mm}$ (length in cutting direction) $\times$ $20\,\mathrm{mm}$ (height h). Insets: surface images of samples *post* cutting onset.

4. Tensile characterization of the gelatin hydrogel, elastomer, and meat-based food material

Characterization of the three materials investigated in the main manuscript via uniaxial tensile tests. Figure S5 contains the experimental results and the cutting force versus displacement curves predicted by the computational model under virtual tensile loading conditions. Note that only the pre-cutting portions of the curves are shown, as the viscous regularization used in the model nonphysically dampens the debonding response even beyond the point of full damage.

5. Calibration of parameters

The parameters of the computational model, used in the main text to disentangle the physics in soft cutting, are calibrated to reproduce the experimental cutting curves and summarized in Tables 1 and 2 in the main manuscript. We note that, for the elastomer (Sylgard 184), the calibration predicts a shear modulus about half the one observed in tensile experiments, as indicated in Figure S5b. The response in stiffness observed in the tensile experiments agrees with the constitutive behavior determined in a biaxial characterization of the elastomer reported by the authors (1). Overall, the differences may be due to slightly different constitutive behavior in tensile deformation and compression loading conditions, even related to volumetric deformation.

6. Traction-separation law in the cutting surface

The cohesive traction forces on the cohesive surface are modeled using traction–separation laws that ensure complete degradation of the tractions when the material undergoes a separation equal to the width of the cutting tool. This behavior is enforced through the inclusion of an exponential term in the law and by selecting a sufficiently small characteristic opening displacement (see Table 2 in the main manuscript for its value). A plot of the damage variable d as a function of the normal opening displacement of the cohesive surface confirms that it approaches 1 (i.e., full degradation) for an opening $[\![\mathbf{u}]\!]_{\mathrm{n}} = 2a$, with a half the thickness of the cutting tool. This is illustrated in Figure S6.

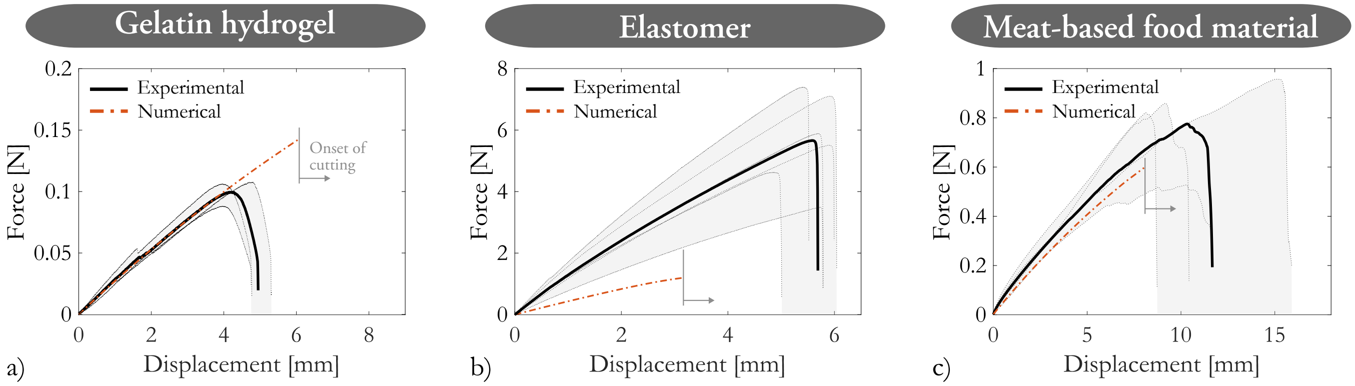

Figure S5. Results for tensile tests on pristine and pre-cut samples and validation of the computational model. Experiments on gelatin hydrogel, elastomer, and meat-based food material. The dimensions of the samples are $12\,\mathrm{mm}$ (width) $\times$ $30\,\mathrm{mm}$ (distance between clamps) $\times$ $3\,\mathrm{mm}$ (thickness). The samples feature an initial notch of $2.4\,\mathrm{mm}$ in the middle of a lateral of the sample. Three experimental repetitions are performed for the same test conditions.

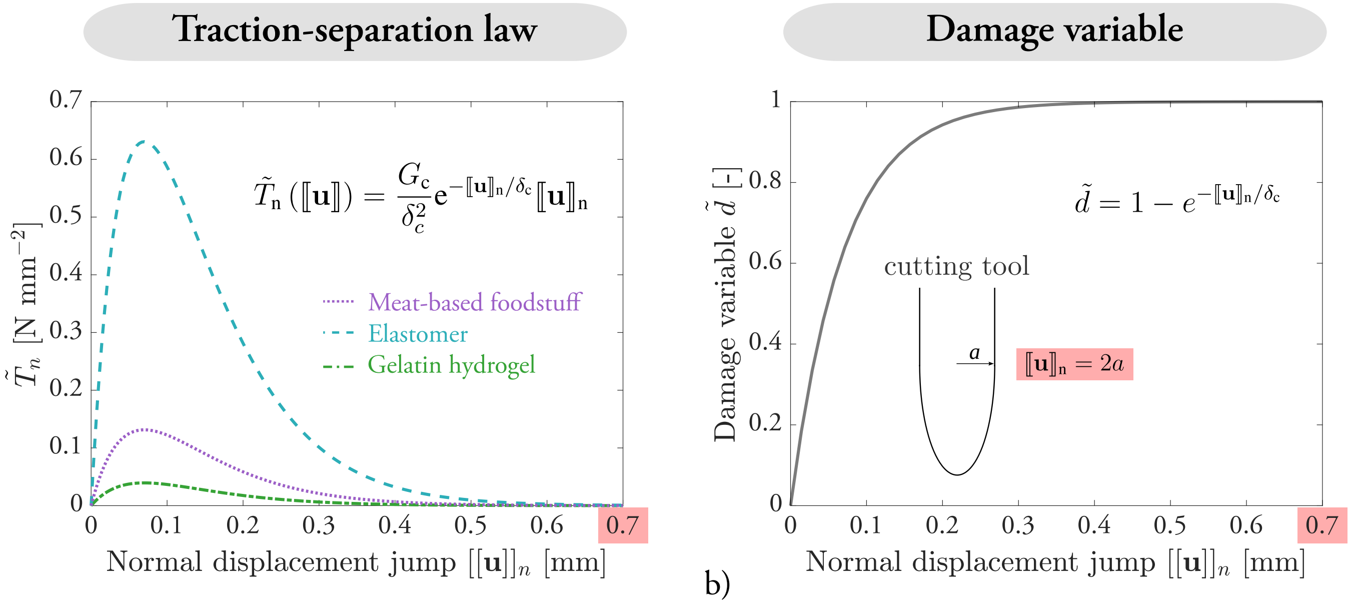

Figure S6. Traction-separation law on the cutting surface. (a) Traction force per unit area as a function of the normal opening displacement across the cutting surface for the three materials: gelatin hydrogel, elastomer, and meat-based food material. (b) Corresponding evolution of the damage variable with respect to the normal opening displacement. When the opening reaches the width of the cutting tool ($\llbracket \mathbf{u} \rrbracket_{\mathrm{n}} = 2a$, see figure inset), the cohesive traction has fully vanished, and the damage variable approaches unity, indicating complete decohesion. For simplicity, the traction–separation law is plotted directly as a function of the normal opening displacement $\llbracket \mathbf{u} \rrbracket_{\mathrm{n}}$ rather than the internal damage variable d (cf. Equation 31 in the main text).

7. Additional cutting simulation for the elastomer material without Coulomb friction ($\mu = 0$)

A simulation with a friction coefficient $\mu = 0$ yields a cutting force, after the onset of cutting, with a slope nearly identical to that obtained when Coulomb friction is included, as shown in Figure S7. This suggests that the tangential resistance arises primarily from adhesion and wear, rather than from Coulomb friction.

8. Principal stress components at the cutting surface along cutting initiation

Figure S8 contains the three principal stress components of the Cauchy stress tensor (σ_1, σ_2, and σ_3) at the cutting surface driving the indentation-to-cutting transition. The principal stresses are arranged according to $\sigma_1 > \sigma_2 > \sigma_3$.

9. Parametric study of critical indentation depth and indentation work at cutting onset for the dimensionless parameter $\frac{G_c/\delta_c}{G}$

Figure S9 shows a parametric analysis of the indentation–to–cutting transition in gelatin hydrogel with varying shear modulus. The study examines the critical indentation depth of the cutting tool at the onset of cutting and the external work performed during indentation up to cutting initiation, both expressed in terms of the dimensionless parameter $\frac{G_c/\delta_c}{G}$. The shear modulus G is systematically varied, while all other parameters are fixed as specified in Table 2 of the main text.

10. Additional cutting experiments on gelatin hydrogel samples to investigate the effect of boundary conditions: width-insensitivity of the Coulomb friction

To investigate the influence of sample geometry on the contact pressure along the cutting surfaces, additional experiments were performed on samples with double the width (perpendicular to the blade) compared to the original ones. The evolution of the cutting force with the displacement of the cutting tool is compared to that of the narrower samples. The results show that increasing the amount of bulk material lateral to the cutting tool has a negligible effect on the cutting force during the cutting regime. The results in Figure S10 indicate that the interfacial mechanics remain largely unchanged, with negligible contact pressure and Coulomb friction.

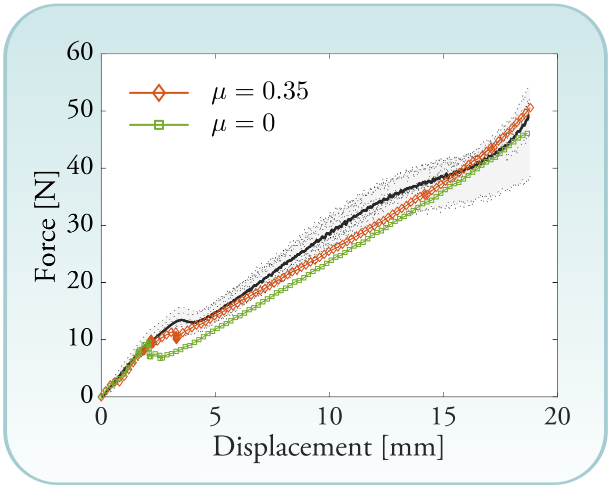

Figure S7. Additional simulation for the elastomer material to assess the effect of Coulomb friction at the indentation-to-cutting transition. To isolate its influence, the friction coefficient is set to $\mu = 0$ and the resulting cutting force–displacement curve is compared against the reference case with active Coulomb friction ($\mu = 0.35$). This comparison reveals the impact of interfacial shear resistance on the force response during the onset of cutting, which smooths the transition.

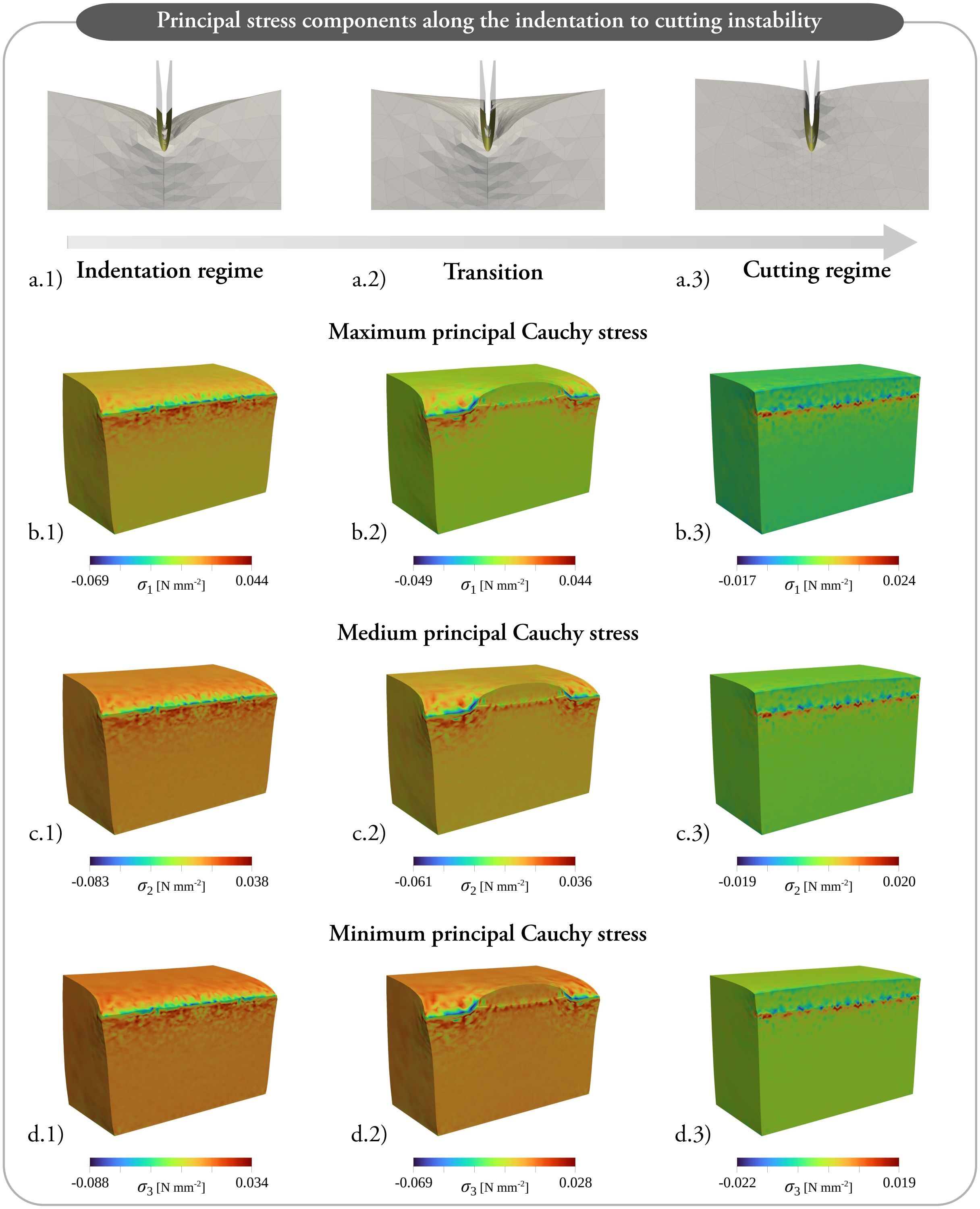

Figure S8. Constitutive behavior on the cutting surface along the unstable indentation-to-cutting transition: principal stress components. (a.1-3) Unstable indentation-to-cutting transition in a gelatin hydrogel at an indentation of the cutting tool where the strain energy of the continuum suffices to overcome the cohesive energy to create a new cutting surface. The onset of cutting occurs at the center of the sample beneath the center of the cutting tool. (b-d) Principal components of the Cauchy stress tensor (σ_1, σ_2, and σ_3) at the cutting surface driving the indentation-to-cutting transition. The principal stresses are arranged according to $\sigma_1 > \sigma_2 > \sigma_3$.

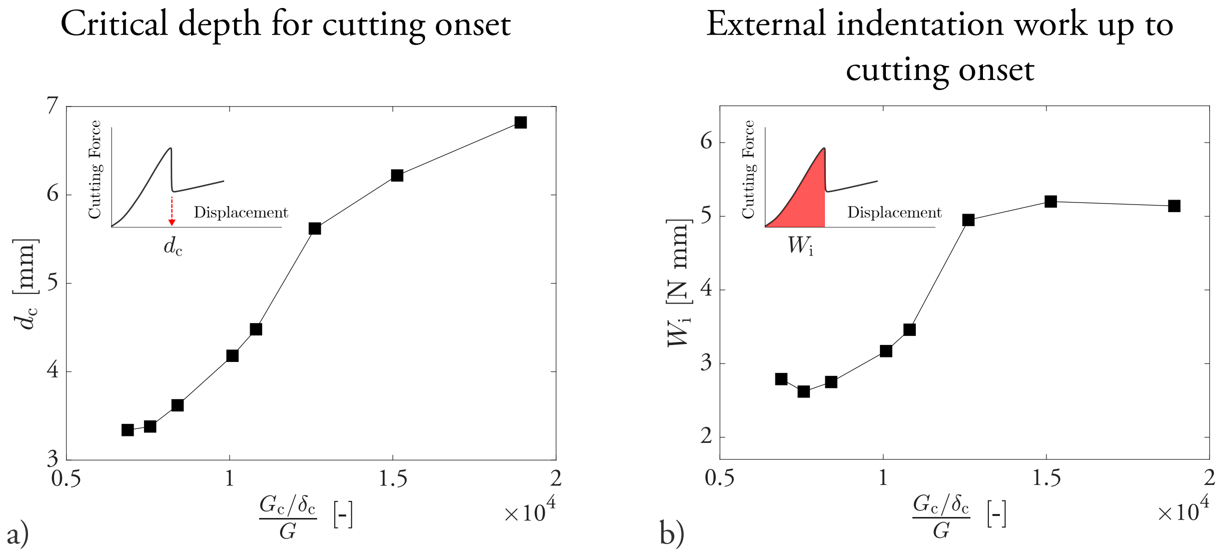

Figure S9. Parametric analysis of the indentation–to–cutting transition in gelatin hydrogel as a function of shear modulus. (a) Critical indentation depth of the cutting tool at the onset of cutting. (b) External work performed by the cutting tool during indentation up to cutting initiation. The analysis is carried out by varying the shear modulus $G \in \{4, 5, 6, 7, 7.5, 9, 10, 11\}$ kPa, while all other material and geometrical parameters correspond to those listed in Table 2 of the main text for the gelatin hydrogel.

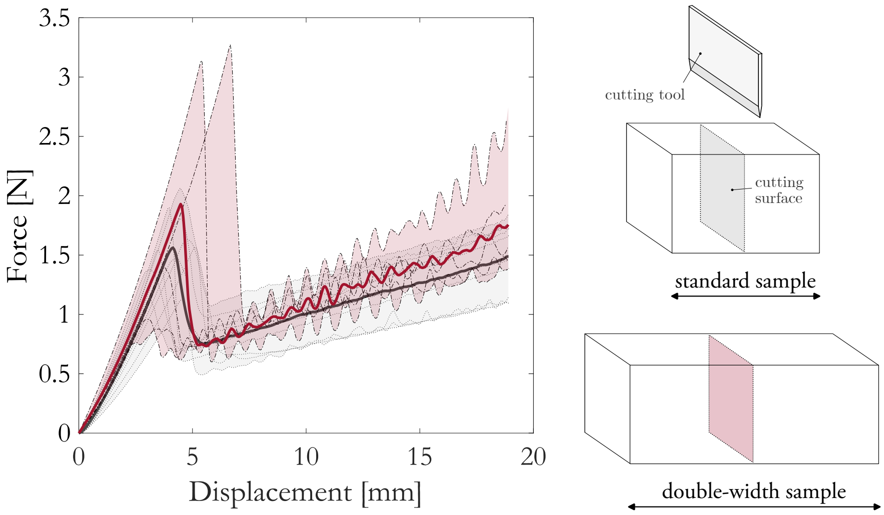

Figure S10. Additional cutting experiments on gelatin hydrogel samples with double width dimension and same cutting length. As revealed by the *in silico* framework in the main text, tangential forces at the tool-material interface are primarily governed by adhesive interactions rather than classical Coulomb friction. This finding is supported by experimental results showing that the slope of the cutting force during steady-state cutting remains nearly unchanged when doubling the sample width from $30\,\mathrm{mm}$ to $60\,\mathrm{mm}$. The insensitivity of the cutting response to lateral bulk volume suggests that steady-state cutting is predominantly controlled by local interfacial mechanisms rather than global material dimensions.

Movie S1. Strain fields evolution during cutting of a gelatin hydrogel sample

Movie S2. Strain fields evolution during cutting of a elastomer sample

Movie S3. Strain fields evolution during cutting of a meat-based food material sample

Movie S4. Animation of the simulated cutting process

References

1. MA Moreno-Mateos, S Wiesheier, A Esmaeili, M Hossain, P Steinmann, Biaxial characterization of soft elastomers: Experiments and data-adaptive configurational forces for fracture. *J. Mech. Phys. Solids* **205**, 106339 (2025).
